# Supplementary material for: RhoGAP domain-containing fusions and PPAPDC1A fusions are recurrent and prognostic in diffuse gastric cancer
Source: Nat Commun. 2018 Oct 25;9:4439. doi: 10.1038/s41467-018-06747-4 (PMC6202325; doi:10.1038/s41467-018-06747-4)
Supplement: Supplementary file 1 — Supplementary Information [file 41467_2018_6747_MOESM1_ESM.pdf]

RhoGAP domain-containing fusions and *PPAPDC1A* fusions are recurrent and prognostic in diffuse gastric cancer

Yang et al.

Supplementary Information

Supplementary Methods

Patients

This study was approved by the National Cancer Center Institutional Review Board (IRB; NCCNCS-120581), and all patients signed IRB-approved consent forms. RNA sequencing analyses were performed in 80 resected tumors and 65 adjacent normal tissue that were collected from early-onset (≤ 45 years) diffuse gastric cancers (DGCs; discovery dataset).

For RT-PCR analysis of gene fusions, the dataset was expanded to 384 biopsy and surgical DGC samples collected in Korea between 2003 and 2017 (Supplementary Table 1). Samples of the expanded dataset were collected from members of National Biobank of Korea (Asan Bio-Resource Center, Keimyung Human Bio-Resource Bank, Biobank of Pusan National University, Biobank of Chonnam National University Hwasun Hospital, Biobank of Chungnam National University Hospital, and Ajou Human Bio-Resource Bank), which is supported by the Ministry of Health and Welfare, from Resource Banks at Dong-A University Medical Center and Kosin University Gospel Hospital and from the National Cancer Center of Korea. Tumors were staged according to the 7th edition of the American Joint Committee on Cancer (AJCC) system.

Biopsy tissue samples were snap-frozen and stored in -80°C freezers until RNA analysis. Resected surgical tissue samples were either immersed in RNAlater (Qiagen, Valencia, CA) or placed in -80°C freezers within 30 minutes after procurement. 2100 Bioanalyzer (Agilent Technologies, Santa Clara, CA) was used to ensure the RNA integrity of frozen resected tumor tissue. To estimate the sample size required for an expanded dataset, we hypothesized that recurrent in-frame fusions are present in 15% of tumors and adversely affect prognosis by a hazard ratio of 2. At two-tailed α and β errors of 0.05 and 0.2, respectively, 128 events were estimated to be required to evaluate the effect of fusions on survival[1]. We assumed that about one third of patients with advanced stage gastric cancers die during 3-year follow-up[2]. For 128 events, therefore, 384 tumors were required as an expanded dataset.

Targeted RNA sequencing analysis was performed on 225 expanded dataset DGCs without available RNA sequencing data. These 225 DGCs were composed of 193 frozen tissue samples and 32 formalin-fixed, paraffin-embedded (FFPE) fragments. Frequencies of in-frame fusions, including *PPAPDC1A* fusions, were determined in a combined dataset of the 225 DGCs and 80 DGCs with available RNA sequencing data (sequenced dataset (n=305); Supplementary Table 2).

RNA sequencing analysis

Transcriptome libraries were prepared using TruSeq mRNA Kit (Illumina, San Diego, CA). From 1–2 μg of total RNA isolated from frozen macrodissected tumors in a discovery dataset, poly(A)+ RNA was

isolated using AMPure XP beads (Beckman Coulter, Brea, CA) and was fragmented using an Ambion Fragmentation Reagents kit (Thermo Fisher Scientific, Waltham, MA). cDNA synthesis, end-repair, A-base addition, and ligation of the Illumina-indexed adapters were performed according to Illumina protocols. Libraries were size-selected for 250–300 bp cDNA fragments on a 3% Nusieve 3:1 (Lonza, Basel, Switzerland) agarose gel, recovered using QIAEX II gel extraction reagents (Qiagen, Valencia, CA), and PCR-amplified using Phusion DNA polymerase (New England Biolabs, Ipswich, MA) for 14 PCR cycles. The amplified libraries were purified using AMPure XP beads. Library quality was measured on an Agilent 2100 Bioanalyzer (Agilent Technologies). Paired-end libraries were sequenced using an Illumina HiSeq 2000 instrument (2 × 100 nucleotide read length). Reads that passed the chastity filter of the Illumina BaseCall software were used for subsequent analyses. RNA sequencing data were aligned at the British Columbia Cancer Agency Genome Sciences Centre. Using BWA aln & sampe (version 0.5.7)[3], all reads were aligned to a human reference genome consisting of hg19/GRCh37-lite and exon-exon junction sequences that were constructed from known transcript models in EnsEMBL, RefSeq and UCSC genes. Default BWA parameters were used for alignments, with the exception of the -s sampe option which was included to disable Smith-Waterman rescue of unmapped mates as this feature was not designed to handle the insert size distribution that occurs in paired-end RNA sequencing data. After BWA alignment, a post-alignment process was performed to reposition the read alignments that spanned across the exon-exon junctions and transform them into large-gapped genomic alignments[4]. The Reads Per Kilobase Of Exon Per Million Mapped Reads (RPKM) values of genes were calculated. GENCODE V3 was used in the quantification process.

Hierarchical clustering and class comparison analyses

We used BRB-ArrayTools (version 4.5.1) for average linkage hierarchical clustering. We used Gene Cluster 3.0 and Java TreeView 1.1.6r4 to display a heatmap after gene centering. To identify differentially expressed genes and pathways, we used BRB-ArrayTools (version 4.5.1). To identify pathways enriched in 3' partner genes of fusions, we used DAVID (https://david.ncifcrf.gov/) gene ontology analysis (GOTerm_BP_Direct).

Hierarchical clustering analysis of the RPKM data revealed four distinct clusters. Cluster 4 was composed of all microsatellite unstable (MSI; n=2) and Epstein-Barr virus (EBV)-positive tumors (n=7). Immune-related genes were overexpressed in Cluster 4 compared with the other Clusters (Supplementary Fig. 1 and Supplementary Tables 3 and 4).

RNA sequencing-based identification of novel gene fusions

To identify gene fusions in early-onset DGC, we used the RnAseq Data Analysis (PRADA)[5,6]. The PRADA pipeline consists of six modules including preprocess, fusion, guess-ft, guess-ig, homology and frame. We first used the preprocess module which can align RNA-Seq reads on human reference genome GRCh37 of NCBI (https://www.ncbi.nlm.nih.gov/projects/genome/assembly/grc/human/) as

well as human transcripts of Ensembl build 64 (http://www.ensembl.org) which allows detection of evidence from both the exon junction and unknown mRNA regions and which generates recalibrated BAM files. We then obtained gene fusion candidates using the fusion module with options of -mm 1 -junL 80 -minmapq 30. The fusion module searches mainly by discordant read pairs, where a read and its pair are mapped to different genes or regions in the genome, and junction spanning reads, where reads span splice junctions and their alignments are split. Finally, we selected the in-frame gene fusion candidates using the prada-frame utility, which is based on the definition of transcript CDS and UTR boundaries from Ensembl, and predicted the functional implication of the potential fusion candidates using the UniProt protein annotation database (http://www.uniprot.org). RT-PCR sequencing analyses were then conducted to validate the expression of 32 candidate fusions that were in-frame and contained partner genes of importance based on the data in the literature (Supplementary Fig. 19).

Additionally, we aligned unmapped reads to hg19 to discover putative breakpoints with deFuse[7], FusionMap[8], and TopHat-Fusion[9]. To remove false-positive breakpoints resulting from these algorithms, we conducted a *de novo* assembly (Trans-ABySS[10] (v1.4.4)) on candidate regions containing putative breakpoints, which were identified by deFuse, FusionMap and TopHat-Fusion, using unmapped reads, split-reads and mapped reads for fusion candidate gene body regions. Selected reads were assembled with ABySS[11] using k-mer 32 to 82. After mapping and assembly, we filtered out fusion candidates that were out-of-frame using an in-house program. As a result, 446 in-frame fusion gene candidates were predicted. We performed RT-PCR sequencing analyses to validate the expression of 182 candidate fusions that were in-frame and contained partner genes of importance based on the data in the literature (Supplementary Fig. 19).

A full list of candidate fusions that were tested by PCR is shown in Supplementary Table 5.

RT-PCR analysis of mRNA breakpoints in a discovery dataset

In-frame fusion candidates were validated for expression using RT-PCR sequencing in a discovery dataset (Supplementary Table 5). cDNA was synthesized from 500 ng of total RNA with SuperScript® III First-Strand Synthesis System (Thermo Fisher Scientific) and amplified using HotStar Taq® DNA Polymerase kit (Qiagen, Valencia, CA). Each PCR amplification was performed using 1 µl of 25 µl cDNA synthesis products. Cycle conditions were: 15 min at 95 °C for initial denaturation, then 40 cycles at 94 °C for 35 sec, 55 °C for 30 sec, and 72 °C for 30 sec, with 10 min at 72 °C for post-extension. For each 10 µl reaction, 300 nM primer, and 0.25 U of HotStarTaq DNA polymerase (Qiagen) were used. For *CLDN18-ARHGPA26,* 5'-GCCTCAGGCCACAGTGTTGC-3' and 5'-CCTTCA CTCTGGCTGTCTTTGTTC-3' were used as primers. For *CTNND1-ARHGAP26,* 5'-TGAGAAGCTGG TGTTGATCAAC-3' and 5'-GAAGCCAATGCTGTCCAACTGC-3' were used as primers. For *ANXA2-MYO9A,* 5'-AGTGCATATGGGTCTGTCAAAGC-3' and 5'-ACTTGGCTGTGGTTTTCAGACAG-3' were used as primers. The other primer sequences were as follows: *TKT-RHOA* 5'-CGGAATCCGCACAAT GACC-3', 5'-GGACAGAAATGCTTGACTTCTG-3', *ZNF292-PREX1* 5'-CGACTACTGTCAGCAGCTGT

G-3’, 5’-GATGTCTTCGATGTTCGAGAACAGG-3’, *ECT2-FABP6* 5’-AGTCAGCAAGGTGGCAAGTTG-3’, 5’-TTTTCGATTACATCGCTGGAGATCC-3’, *EML4-ALK* 5’-CAGGTGGAGTCATGCTTATATG-3’, 5’-CTTGCTCAGCTTGTACTCAG-3’, *PGAP3-VMP1* 5’-TGGCCTCGTTTCTCAATGG-3’, 5’-GAACTCTT CATCATCTGGTTCAG-3’, *TACC2-PPAPDC1A* 5’-GCCAGGTCTCTACGGATCTG-3’, 5’-GGTAGGTAT GTTATCTGATTGCAC-3’, 5’-ATGGGCAATGAGAACAGCAC-3’, 5’-GTAGGTATGTTATCTGATTGCAC-3’, *LONP1-SAFB* 5’-AGGACGTCCTGGAAGAGACC-3’, 5’-CACGCTACTTGTGTCCTGTG-3’, *LUC7L3-C10orf76* 5’-CGTAGGATCAGACGAGGCCATG-3’, 5’-GGACGTTGGTGCTAATGGATCG-3’, *CLSTN1-EFCAB7* 5’-CACCTACCACGGCATAGTCACAG-3’, 5’-TGCATAGATATCAATGACAAATGGA CATG-3’, *ARFGAP2-SLC1A2* 5’-ATGTTGGTACTTTCGCCTCTGGAC-3’, 5’-CCACAACATTGACTGA AGTTCTCATCC-3’, *TERF2-CDH3* 5’-CTTCAGCGCCACCATCCAAG-3’, 5’-ATCTTCCGCTTCTTTCT CACCAAC-3’, *GTF2I-FBF1* 5’-TGATGGTAACAGATGCTGACAGGTC-3’, 5’-ATGGTGCTGAAGACAT CATCACC-3’, *ARMC7-PEX14* 5’-CTGGTCACGGAATTCCAGGAGAC-3’, 5’-CAGCTGCTTTCTGTCC TCTC-3’, *IFFO2-UBR4* 5’-AACGTGCTGGCCAAGGTGAAG-3’, 5’-GCCTGTACATTCCTGGAGGTAAG TG-3’, *INTS12-TBCK* 5’-AGATGGGATTGGCCTGCGTTG-3’, 5’-CAAGTAGTAAGGTATCCCAGAGGT GG-3’, *UBE2L3-MAPK1* 5’-CGTAACATCCAGGTTGATGAAG-3’, 5’-GAATGGTGCTTCGGGGATG-3’, *ELK3-NTN4* 5’-CGATGGTGAATTCAAGCTCCTC-3’, 5’-ATGGCCGGTCATTGTATAACG-3’, *EIF4G2-UPK2* 5’-GTGCACCTCAGCACTATCC-3’, 5’-CTCTCTGCTGGACTCAGTG-3’, *RICTOR-GHR* 5’-GAA TGACAGCGGCGAGGAG-3’, 5’-AGGCCTGGATTAACACTTTGC-3’, *RNASEH2C-CFL1* 5’-GACTGAC GACCAAGAGGAGGAG-3’, 5’-GAAGACTTACGCACCTTCATGTCG-3’, *ARHGAP26-NDFIP1* 5’-CTG CACACGGCGGAAAACA-3’, 5’-TGGGCAGTGTTGTAG CTACATTG-3’, *IDUA-GAK* 5’-CTTCTGGAGG AGCACAGGCTTC-3’, 5’-AGCATCATACTGAGCCGTGG-3’, *ARIH1-PPAPDC1A*, 5’-ACGATGATACCC TGGATCTGG-3’, 5’-AGTGTTTGTGCAGACTCCATTC-3’, *SHTN1-PPAPDC1A*, 5’-GAGGCAAGCAGT TGAAGAGA-3’, 5’-TCGCAGACTAACGTAGGGTT-3’, *FGFR2-TACC2*, 5’-CAAGTGGATGGCTCCAGA AG-3’, 5’-ATCATCTGAGCGATGGTCTTC-3’, 5’-CAAGTGGATGGCTCCAGAAG-3’, 5’-ACTGTCTCAG AACCACATCCT-3’, *SEC23IP-TACC2*, 5’-GAAGCACTTAGCCTCTCTGAAT-3’, 5’-ATCATCTGAGCGA TGGTCTTC-3’, *NSMCE4A-TACC2*, 5’-AAGCCACGAGTTGATCGTC-3’, 5’-ACTGTCTCAGAACCACA TCCT-3’, *TACC2-WDR11*, 5’-CTACACCAGAAACACCACCAG-3’, 5’-CCACTGGTGAGATGGTACAC-3’, *VMP1-RPS6KB1*, 5’-CATTCAGCAAGCACATAGTGGA-3’, 5’-TCCCAGTATTTGCTCCTGTTAC-3’, *ARHGAP26-CAST*, 5’-CTACGTGCAGGAGAAACGTC-3’, 5’-CAGGAGTGGCTTTCCATCTT-3’, *CLDN18-ARHGAP6*, 5’-GCCTCAGGCCACAGTGTTGC-3’, 5’-CCCTGTCATTCGCAATGACT-3’, *PGAP3-PSMD3*, 5’-GGGACGACTGTAAGTATGAGTG-3’, 5’-GTGTAGGTAATTCCGCAGCA-3’, *PGAP3-PIP4K2B*, 5’-GGGACGACTGTAAGTATGAGTG-3’, 5’-GCCATAGGAGCAGAGTAGGT-3’, *RICTOR-C5orf28*, 5’-GAAGAACCTCCGAGTACGAGG-3’, 5’-TGGGCATATCCACAAACCAT-3’, *NFAT5-TERF2*, 5’-CGGCCTTGATCCTAGCAAC-3’, 5’-ATAGCTGATTCCAGTGGTGTG-3’, *RHOA-USP4*, 5’-TTCGTTGCCTGAGCAATGG-3’, 5’-GACTGCAAGGTCTGCCTG-3’, *ECT2-FNDC3B*, 5’-ACCAGTACAGAGGTTACCCAG-3’, 5’-GCTTGCTGTACTGGTCTTCT-3’, *ARHGAP26-GLRA1*, 5’-CTACGTGCAGGAGAAACGTC-3’, 5’-GATGCTGTAGAGGACATTCCC-3’

RT-PCR analysis of mRNA breakpoints in an expanded dataset

Total RNA samples from the expanded dataset were subjected to RT-PCR analyses of 25 validated in-frame fusion using RT-PCR methods described above. Synthesized cDNA was PCR-amplified for 35 cycles.

Exon-level expression

To quantify mRNA expression in exon level, the Fragments Per Kilobase Of Exon Per Million Fragments Mapped (FPKM) values of exons and genes were calculated using by RSEM[12].

RhoA pull-down activation assay and mutation analyses

RhoA pull-down activation assays (RhoTekin assay) were performed using a RHOA Activation Assay Biochem Kit (BK036; Cytoskeleton, Denver, CO) as recommended by the manufacturer, with some modifications. Cells were harvested and lysed using T-PER reagent with protease inhibitors. After protein concentration was measured using a BCA reagent, pull-down was performed using 0.3 mg of total protein and 60 µg of Rhotekin-RBD beads in 0.5 mL of T-PER Reagent at 4°C for 1 h. The bead pellets were washed twice with T-PER Reagent that was diluted 1:10 in PBS. After the last wash, buffer was removed leaving 90 µl of buffer and the bead mixture, 30 µl of 4x Laemmli sample buffer was added to the remaining mixture, and the samples were boiled for 5 min. Twenty microliter of the sample volume was loaded for Western blot analysis. Band intensity was quantified using ImageJ software and normalized to total RHOA protein.

Mutations in *CDH1, RHOA* and *TP53* were identified by targeted DNA sequencing analyses.

Cell lines

All cells were grown in RPMI-1640 with 10% fetal bovine serum and 1x ZellShield (13-0050, Minerva Biolabs GmbH, Berlin, Germany). NCC-S1 and NCC-S1M cell lines were established by our group from a diffuse-type gastric adenocarcinoma formed in a *Villin-cre; Smad4*$^{F/F}$*; Trp53*$^{F/F}$*; Cdh1*$^{F/+}$ mouse. *Pdx1-cre; Smad4*$^{F/F}$*; Trp53*$^{F/F}$*; Cdh1*$^{F/+}$ cells were primary cultured by our group from a diffuse-type gastric adenocarcinoma formed in a *Pdx1-cre; Smad4*$^{F/F}$*; Trp53*$^{F/F}$*; Cdh1*$^{F/+}$ mouse. NUGC4 cell line was purchased for this work from RIKEN Cell Bank (Tsukuba, Japan). SNU-638 and SNU-719 were validated for the identity by STR DNA profiling. ImSt, a conditionally-immortalized, mouse gastric epithelial cell line[13], was grown at 33°C. All cell lines were routinely tested and confirmed to be free of Mycoplasma contamination using a Mycoplasma PCR with positive and negative controls. For generation of stable cell lines, three days after 293FT cells were transfected with one of the cloned gene expression vectors, pMD2.G (Addgene), and psPAX2 (Addgene), the growth medium was harvested and filtered using 0.45 µm membrane. To generate a *TACC2-PPAPDC1A* lentiviral vector, *TACC2* coding sequence 1-146 and *PPAPDC1A* coding sequence 57-816 were ligated and cloned using a pCDH-CMV-MSC-EF1-Puromycin vector. To validate transgene expression, cDNA was synthesized from 1 µg of total RNA using SuperScript III (18080-051, Thermo Fisher Scientific), and

PCR amplifications were performed for 45 cycles using 1 µl of synthesized cDNA per 5 µl of the 2x QuantiFast SYBR Green PCR Master Mix (204054, QIAGEN) and the following primers: *CLDN18-ARHGAP26*, 5'-GCCTCAGGCCACAGTGTT GC-3' and 5'-CCTTCACTCTGGCTGTCTTTGTTC-3'; *Myo9a*, 5’-GTCAGCTCCTCCGTTTCTT-3’ and 5’-CTGAAGCAGCTCGGTAAAT-3’; *GAPDH*, 5'-GAGTCAACGGATTTGGTCG-3' and 5'-TGGAATCATATTGGAACATGTAAAC-3' (Supplementary Figs. 6 and 16).

Whole genome sequencing analysis

To locate chromosomal breakpoints, whole genome sequencing (WGS) analyses were conducted using by HiSeq platforms at a median depth of 32.6x. The adapter sequence was removed and paired reads were selected using by Trimmomatic. All reads were aligned to human reference sequence (hg19) by BWA-MEM. Structural variation in genome was identified by SvABA, which is identification method for structural variants with String Graph Assembler algorithm. WGS-identified, genomic DNA breakpoints were confirmed by PCR capillary sequencing analyses analyses (Supplementary Table 8). For *ANXA2-MYO9A*, 5’-TCCATACAACAGAATATTTGGC-3’ and 5’-TGTGGATGAGGTCACCAT-3’ were used as primers.

Targeted DNA sequencing analysis

Targeted DNA sequencing analysis was performed with a mean coverage of 960x on samples from 229 DGC samples for *CDH1*, *TP53, ARID1A, KRAS, PIK3CA, ERBB3, TGFBR1, FBXW7, RHOA,* and *MAP2K1*[14]. Using the Ion AmpliSeq Designer software, PCR primers were designed for all exons (with 5 bp of padding at the ends of ends) with 98.9% coverage. PCR amplicons ranged from 125 to 175 bp in length. A total of 20 ng of genomic DNA with A260/A280 and A260/A230 ratios >1.6 was used for library generation. Fragment libraries were constructed using DNA fragmentation, barcode and adaptor ligation, and library amplification using an Ion DNA Barcoding kit (Thermo Fisher Scientific, Carlsbad, CA), in accordance with the manufacturer’s instructions. The size distribution of the DNA fragments was analyzed using a 2100 Bioanalyzer High Sensitivity Kit (Agilent Technologies). Template preparation, emulsion PCR, and ion sphere particle (ISP) enrichment were performed using an Ion Xpress Template OT2 200 kit (v3: 4488318, Thermo Fisher Scientific), in accordance with the manufacturer’s instructions. ISPs were loaded onto a P1 chip (v2) and sequenced using an Ion P1 sequencing 200 kit (v3: 4488315, Thermo Fisher Scientific). Ion Torrent platform-specific pipeline software (Torrent Suite v4.4) was used to separate the barcoded reads, generate sequence alignments with the hg19 human genome reference, perform target-region coverage analysis, and filter and remove poor signal reads. The single-nucleotide variants and small insertions/deletions (indels) were compared with those in the germline genomic DNA. Initial variant calling was generated using Torrent Suite with a plug-in program (Variant Caller v4.4). The alignment file from the Torrent Suite was then transferred to Ion Reporter v4.4 to generate a somatic variant file using default parameters. The somatic calls generated from Ion Reporter v4.4 were further filtered using the

following criteria: 1) > 50 reads in tumor samples; 2) > 5 somatic variant reads; 3) somatic variant allele frequencies > 0.05 and > 0.1 for SNV and indels, respectively; and 4) minor allele frequency < 0.02 in germline samples.

TCGA subgroup assignment

We categorized 200 DGCs into one of four subtypes (EBV-positive (EBV), MSI-high (MSI-H), genomically stable (GS) and chromosomal instability (CIN))[14]. CIN-positive tumors without EBV or MSI were assigned to the CIN subgroup, and CIN-negative tumors without EBV or MSI were assigned to the GS subgroup. If a tumor was both EBV-positive and CIN, that sample was assigned to the EBV group. EBV status was determined by PCR. The PCR mixture (10 μl) contained 0.25 units of HotStarTaq DNA Polymerase (Qiagen), 1 μl of 10x PCR Buffer, 200 μM of each dNTP, 0.3 μM of each primer, and 5 ng of genomic DNA from the tumor sample. The primer sequences were 5'-CCATGTAAGCCTGCCTCGAG-3' and 5'-GCCTTAGATCTGGCTCTTTG-3', and the cycling conditions were 95°C for 15 min followed by 30 cycles of 94°C for 40 s, 57°C for 30 s, and 72°C for 30 s, and a final extension of 72°C for 3 min. CIN status was determined based on Affymetrix SNP6.0 data[14]. SNP6.0 data were subjected to the segmentation using the Circular Binary Segmentation method, and were analyzed using hierarchical clustering along with TCGA tumors whose CIN statuses were previously defined. The CIN status of each tumor was determined based on the cluster membership.

Statistical analyses

We used SAS 9.3 (SAS Institute Inc., Cary, NC) for survival analysis. Log rank tests were used to determine the significance of difference in survival times between groups. Cox proportional hazards model was used for multiple regression analyses of 172 DGCs with available SNP6.0 array and targeted DNA sequencing data.

Chi-square test was conducted to evaluate the chromosomal distribution of in-frame fusions that were identified by targeted RNA sequencing analyses. We assumed the equivalent hybridization capture performance across probes in our custom targeted RNA sequencing panel. Expected frequencies of in-frame fusions involving a given chromosome arm were determined based on the total length of genes represented by probes located at the corresponding chromosome arm.

Western blot analysis

Cells were lysed using T-PER Tissue Protein Extraction Reagent (Thermo Fisher Scientific) containing protease and phosphatase inhibitors. Protein was quantified using a BCA Protein Assay Kit (Thermo Fisher Scientific). Boiled protein samples were separated by SDS-PAGE and blotted onto nitrocellulose membranes. After blocking using 2.5% bovine serum albumin in PBS with Tween20 (PBS-T; Sigma-Aldrich, St. Louis, MO), immunoblotting was conducted with primary antibodies (1:1,000) in 5% BSA in PBS-T overnight, followed by a horseradish peroxidase-conjugated secondary antibody (anti-rabbit IgG; GenDEPOT, Barker, TX) (1:5,000) for 1 h. Anti-MYO9A (HPA039812,

Sigma-Aldrich), anti-RHOA (#2117, Cell Signaling, Danvers, MA) and anti-Vimentin (ab8978, Abcam, Cambridge, MA) were used as primary antibodies. SuperSignal West Pico Chemiluminescent Substrate kit (Thermo Fisher Scientific) was used for detection. Chemiluminescence images were taken using FUSION SOLO (Vilber Lourmat, Eberhardzell, Germany).

***In vitro* slow aggregation assays**

After the cells were detached using trypsin, 2 x 10^4 cells in 0.2 ml of growth medium were seeded into 96-well plates that were pre-coated with 45 µl of 0.8% DIFCO Noble Agar (BD Biosciences, San Jose, CA) in PBS. After 24 h of incubation, the cell aggregation was evaluated under a light microscope (50x) (Axio Observer Z1, Carl Zeiss, Jena, Germany).

***In vitro* proliferation assay**

NCC-S1, NCC-S1M, and *Pdx1-cre;Smad4$^{F/F}$;Trp53$^{F/F}$;Cdh1$^{F/+}$* cells were seeded in 96-well plates at 2–5 × 10^3 cells/well in a final volume of 200 µl. After 1, 2, and 3 days in a humidified incubator at 37°C with 5% CO_2, 20 µl MTT (3-(4,5-Dimethyl-2-thiazolyl)-2,5-diphenyl-2H-tetrazolium bromide (M2003, Sigma-Aldrich) was added to each well. The ImSt cell line was seeded in 96-well plates at 10^4 cells/well in a final volume of 200 µl. After 1, 2, and 3 days in a humidified incubator at 37°C with 5% CO_2, 20 µl MTS (G5421, Promega, Madison, WI) was added to each well. ImSt cells were also seeded in 24-well plates at 2 × 10^4 cells/well in a final volume of 1 ml. After 1 and 2 days in a humidified incubator at 37°C with 5% CO_2, 100 µl of MTS was added to each well. Optical densities were measured by a microplate reader at 570 nm and 490 nm, for MTT and MTS assays respectively. Ectopic expression of *TACC2-PPAPDC1A* very modestly promoted the proliferation of ImSt cells (Supplementary Fig. 18).

Sphere formation and soft agar colony formation assays

For sphere formation assays, trypsinized cells were plated at 2 ×10^3 cell per well in serum-free DMEM/F12 media (10-090-CVR, Corning, Corning, NY), supplemented with 10 ng/ml fibroblast growth factor (GF003, Millipore, Burlington, MA), 20 ng/ml epidermal growth factor (PHG0311, Thermo Fisher Scientific), in low attachment 24-well plate for 7 days. For colony formation assay in soft agar, trypsinized cells were seeded at 1 × 10^5 cell per well in 1 ml of media and 3.5% low-melting temperature Noble Agar (BD Biosciences) in low-attachment 6-well plates coated with 1 ml of 5% low-melting temperature Noble Agar (BD Biosciences).

Migration and invasion assays

For migration assays, cells were harvested using trypsin, and were placed on 24-well inserts with 8 µm pore (353097, BD Biosciences) in serum-free RPMI-1640 media. We used 2 x 10^4 mouse or 2 x 10^5 human gastric cancer cells per well. For invasion assays, 3 x 10^5 trypsinized cells per well were placed on 24-well Matrigel inserts with 8 µm pore (354480, BD) in serum-free RPMI-1640 media. For

both assays, medium containing 10% FBS was subsequently added to 24-well insert plate (354578, BD Biosciences) and cells were cultured at 37°C under 5% CO_2. At 24 h after plating, remaining cells were removed by gentle scrapping of the upper chamber with a wet cotton swab, fixed with 10% formalin for 20 min and washed with PBS once. The inserts were soaked in hematoxylin for 5 min and washed. Membranes were cut from inserts and moved to a glass slide. Average number of invaded cells was determined after counting 5 high power fields (100x). Number of migrated cells expressing *CLDN18-ARHGAP26* was normalized to migrated cells expressing an empty vector for each experiment.

***In vivo* tumorigenicity and growth assays**

Each mouse cell line (10^7 NCC-S1 cells, 10^6 *Pdx1-cre; Smad4*$^{F/F}$*; Trp53*$^{F/F}$*; Cdh1*$^{F/+}$ cells, and 10^5 NCC-S1M cells) that stably expressed either empty vectors or *CLDN18-ARHGAP26* fusion vectors was injected into the subcutaneous tissue of the flank of 7-week-old BALB/c nude mice (*CAnN.Cg-Foxn1nu/Crlj*, n=5 per group). Tumorigenicity was determined after 5 days. Tumor volume was then measured two times a week using a caliper and the tumor volume (in mm^3) was calculated by the formula of volume = $(\text{width})^2$ x length/2. This study was reviewed and approved by the Institutional Animal Care and Use Committee of the National Cancer Center Research Institute (NCCRI). The NCCRI is accredited by the Association for Assessment and Accreditation of Laboratory Animal Care International and abides by the guidelines outlined by the Institute of Laboratory Animal Resources.

Immunohistochemistry (IHC)

Gastric cancer tissues were fixed in 10% phosphate-buffered formalin and embedded in paraffin. IHC was performed on 4 μm-thick serial sections from whole-tissue paraffin blocks. For MYO9A IHC staining, antigens were retrieved from sections using heat treatment for 15 min in Tris-EDTA buffer (pH 8.0, ab93680, Abcam) at 95°C. Endogenous peroxidases were blocked with 3% H_2O_2 (878973, Thermo Fisher Scientific) for 10 min at room temperature. For the blocking of nonspecific binding, a ready-to-use protein blocker solution (X0909, DAKO, Santa Clara, CA) was added for 20 min at 37°C. Sections were then incubated with a primary antibody against MYO9A (2 μg/ml, HPA039812, Sigma-Aldrich) overnight at 4°C. Afterwards, the sections were incubated with a horseradish peroxidase polymer-conjugated secondary antibody for 20 min at 37°C and stained using DAB chromogen for 3 min (Real Envision detection system, K5007, DAKO). A hematoxylin counterstain was then applied. Cytoplasmic staining and membrane staining were evaluated and graded as either grade 0 (negative), grade 1 (weakly positive), or grade 2 (strongly positive). Grade 1 immunostaining was defined as MYO9A immunostaining equivalent to normal gastric foveolar epithelium. Grade 2 immunostaining was defined as MYO9A immunostaining that was unequivocally stronger than immunostaining in normal gastric foveolar epithelium.

Phosphoproteome profiling analyses of mouse gastric cancer cell lines

Protein lysate was obtained from NCC-S1 cells expressing an empty vector, NCC-S1 cells expressing *CLDN18-ARHGAP26*, NCC-S1M cells expressing an empty vector, NCC-S1M cells expressing *CLDN18-ARHGAP26*, *Pdx1-cre;Smad4*$^{F/F}$*;Trp53*$^{F/F}$*;Cdh1*$^{F/+}$ cells expressing an empty vector, and *Pdx1-cre;Smad4*$^{F/F}$*;Trp53*$^{F/F}$*;Cdh1*$^{F/+}$ cells expressing *CLDN18-ARHGAP26*, at 70-80% confluence. After washing with cold PBS three times, we scraped mouse gastric cancer cells from 150mm culture dishes using a cell scraper with 8-10 mL of PBS. Scraped cells were lyzed in 100mM Tris-HCl buffer (pH 7.6) with 4% SDS and the protease inhibitor cocktail tablet (Roche, Basel, Switzerland). The Sonicator 3000 (Misonix, Farmingdale, NY) was then used with power level 5 (pulse-on for 15 sec and pulse-off for 10 sec for 12 times). Lysate was then centrifuged at 14,000 g at 4 °C for 15 min. Microcon-30kDa centrifugal filter unit with ultracel-30 membrane (Merck Millipore, Burlington, MA) was used for the filter-aided sample preparation (FASP).

Each 250 μg of peptide sample was labeled with 10-plex TMT reagent (Thermo Fisher Scientific). All of the TMT labeled peptides were pooled and dried with vacuum centrifugation. We conducted a reverse-phase liquid chromatography to fractionate pooled TMT-labeled peptide samples using the 1260 Infinity HPLC system (Agilent Technologies). We used the Xbridge C18 analytical column (4.6 mm × 250 mm, 130 A, 5 μm) for the peptide separation. We used 10 mM triethylammonium bicarbonate (TEAB) in water (pH 7.5) and 10 mM TEAB in 90 % acetonitrile (pH 7.5), respectively, for solvents A and B. We fractionated peptides using a 115 min-gradient at a flow rate of 500 μL/min [0 % solvent B (10 min), 0→5% solvent B (10 min), 5→35% solvent B (60 min), 35→70% solvent B (15 min), 70% solvent B (10 min), and 70→0% solvent B (10 min)]. Ninety-six 1-min fraction segments (15-110 minutes) were pooled into 12 non-contiguously concatenated peptide fractions, dried, and stored at -80 °C.

Magnetic $Fe_3{}^+$-NTA-agarose beads were freshly prepared using Ni-NTA-agarose beads (QIAGEN, Hilden, Germany) for phosphopeptide enrichment. Total peptide was reconstituted in 500 μL of IMAC binding/wash buffer (80% acetonitrile, 0.1% formic acid) of each fraction, and incubated in 100 μL of 5% bead suspension with end-over-end rotation at RT for 30 min. After incubation, beads were washed 3 times each with 500 μL of wash buffer. Phosphopeptides were eluted from the beads using 125 μL of 1:1 (acetonitrile: 2.5% ammonia in 2 mM phosphate buffer (pH 6.5)) mixed buffer (pH 10.0) after incubating at RT for 1.5 min. Then, samples were acidified to ~pH 3.5 and concentrated to 5-10 μL, and were reconstituted to 45 μL with 10% trifluoroacetic acid (TFA) for LC-MS/MS analysis.

We injected each of the 12 peptide fractions into the Ultimate 3000 HPLC system (Thermo Fisher Scientific, San Jose, CA) that was online-coupled to Q-Exactive Orbitrap mass spectrometer (Thermo Fisher Scientific, San Jose, CA). EASY-spray LC columns (2 μm, 500 x 0.075 mm) and PepMap C18 LC columns (3 μm, 20 x 0.075 mm) were used capillary analytical column and a solid phase extraction (SPE) column, respectively, in Ultimate 3000 HPLC systems. The capillary analytical column temperature was kept at 60 °C. We used 0.1 % formic acid in water and 0.1 % formic acid in acetonitrile, respectively, for solvents A and B, and a 180 min-gradient (5 → 10% solvent B over 10 min, 10 → 30 % over 150 min, 20 → 40 % solvent B over 20 min, 80 % solvent B for 10 min and 5 % solvent B for 15

min) for profiling. We set the flow rate and the desolvation capillary temperature at 300 nL/min and 250 °C, respectively. We acquired full MS scans for the mass range of 400–2000 Th at a resolution of 70,000. We fragmented 12 most abundant ions by data-dependent MS/MS experiments with an isolation window of ± 0.8 Th, an exclusion duration of 30 s, and at a normalized collision energy of 30 for higher-energy collisional dissociation. We acquired the MS/MS scans at a resolution of 35,000 with a fixed first m/z of 100 Th. The maximum ion injection time was 120 ms for full MS and MS/MS scan. The automatic gain control target value was set at 1.0×10^6 for both MS and MS/MS scans.

All of LC-MS/MS data were mapped using Proteome Discoverer (v 1.4) software (Thermo Fisher Scientific, San Jose, CA) against the mouse UniProt database containing 16,958 protein sequence entries (2018.02.18). We allowed semi-tryptic cleavage, and set the precursor mass tolerance at 10 ppm. Carbamidomethylation of cysteine (+57.0215 Da) and TMT modifications at N-terminus of the peptide and lysine (+229.1629 Da) were set as static modifications. Oxidation to methionine (+15.9949 Da) and phosphorylation of serine, threonine, and tyrosine (+79.9663 Da) were set as a variable modification. All resulting spectral identification files were validated by Scaffold Q+ (v 4.8.4). All spectra were filtered out by follow parameters; protein threshold > 99%, peptide threshold > 95%, a minimum of 2 unique peptides, and phosphorylated peptides. Reporter ion intensities of TMT label tags were extracted by quantitation module in Scaffold Q+ (v 4.8.4).

We identified and quantified 7,944 non-redundant phosphoproteins. For each phosphoprotein, we calculated reporter ion intensity ratios between cells overexpressing *CLDN18-ARHGAP26* and those expressing an empty vector. We then determined the average reporter ion intensity ratio (between cells overexpressing *CLDN18-ARHGAP26* and those expressing an empty vector) for each phosphoprotein among NCC-S1 cells, NCC-S1M cells, and *Pdx1-cre;Smad4*$^{F/F}$*;Trp53*$^{F/F}$*;Cdh1*$^{F/+}$ cells. Phosphoproteins were then ranked by the average reporter ion intensity ratio. DAVID pathway analysis (https://david.ncifcrf.gov) was performed to identify pathways enriched in top 500 phosphoproteins that were overexpressed by *CLDN18-ARHGAP26.*

Detection of *H. pylori* using real-time PCR

H. pylori status was evaluated by real-time PCR using a LightCycler 480 (Roche Diagnostics, Basel, Switzerland) with 8 μl of reaction mixture containing 4 μl of 2x QuantiTect SYBR Green PCR Master Mix (Qiagen), 250 nM of each primer, and 5 ng of genomic DNA[14]. The cycling conditions were as follows: 95°C for 15 min, followed by 45 cycles of 95°C for 20 s, 58°C for 20 s, and 72°C for 20 s. The primers used were Hp23S 1835F (5'-GGTCTCAGCAAAGAGTCCCT-3') and Hp23S 2327R (5'-CCC CCAAGCATTGTCCT-3'). Cp value < 40 was interpreted as a positive result. In 70 patients (21.5%), *H. pylori* status was evaluated by the H/E stain of resected tumor tissue. Overall, *H. pylori* was present in 40.5% of samples tested (132 of 326; Supplementary Table 9).

Targeted RNA sequencing

Our custom targeted RNA sequencing panel used hybrid capture probes (Agilent Technologies) for

capturing all exons of 25 in-frame fusions identified in our initial RNA sequencing analysis of the discovery set (Table 1). Using 200 ng of total RNA, cDNA was prepared for library construction with the SureSelect RNA Direct (Agilent Technologies). Captured libraries were loaded into the HiSeq 2500 according to the manufacturer's recommendations (Illumina). Raw image files were processed by HCS1.4.8 for base-calling with default parameters and sequences of each individual were generated as 101-bp paired-end reads. Sequences were mapped against the human reference genome (Ensembl release 72) using TopHat v2.0.9 with default options for paired-end sequences. Fusion-gene discovery was performed using deFuse with default parameters.

Among 304 expanded dataset DGCs without available RNA sequencing data, 225 DGCs (193 frozen tissue and 32 FFPE fragments) were subjected to targeted RNA sequencing analyses. The mean sequencing coverage was 56.1-fold (range, 17.1–117.4) and the mean read count was 26,628,138 (range, 8,099,628–55,715,640). In-frame fusion transcripts with > 3 spanning reads were identified as gene fusions (Table 2), and read-through transcripts were excluded. All in-frame fusion candidates were validated by capillary RT-PCR sequencing.

Lipidomic profiling analysis[15]

Standard phosphatidic acid (PA) (10:0–10:0), lysophosphatidic acid (LPA) (C17:0), and diacylglycerol (DG) (8:0–8:0) were purchased from Larodan Fine Chemicals AB (Malmö, Sweden). Each lipid standard was dissolved in chloroform/methanol (1:1, v/v) and stored at -20°C. The early-onset DGC expressing *TACC2-PPAPDC1A* (n=1) and eight randomly-selected DGCs without the fusion were analyzed. For each tumor, macrodissected cancer tissue (0.5-2.5 mg dry weight) was mixed with 50 µl lipid standards [DG (5 ng/ µl), PA (6 ng/ µl), and LPA (6 ng/µl)], 610 µl methanol, and 330 µl chloroform. The mixture was sonicated for 10 min, and centrifuged for 2 min at 14,000 x g and 4'C. Supernatants were then vacuum-dried and reconstituted in 60 µl methanol. PA and LPA were subjected to TMSD methylation. A 2 M solution of TMSD in 20 µ hexane was added to the lipid extracts resuspended in 20 µl of methanol. After vortexing for 30 s, methylation was performed at 37°C for 15 min. Addition of 0.5 µl glacial acetic acid quenched the methylation for subsequent analysis. HPLC analysis was performed using a 1290 Infinity series HPLC instrument (Agilent Technologies). A Hypersil GOLD column (2.1×100 mm ID; 1.9 µm, Thermo Fisher Scientific) was used for lipid separation. Temperatures of the column oven and sample tray were adjusted to 40°C and 4°C, respectively. Solvent A consisted of an acetonitrile/methanol/water mixture (19:19:2) with 0.1% (v/v) formic acid and 20 mM ammonium formate, and solvent B consisted of 2-propanol with 0.1% (v/v) formic acid and 20 mM ammonium formate. The flow rate was 0.25 mL/ min and the injection volume was 2 µl for each run. A 30-min lipid elution gradient was performed as follows: during the first 5 min, solvent composition was set at 95% A and 5% B; and a first linear gradient to 70% A and 30% B for 10 min; was followed by a second gradient of 5% A and 95% B for 7 min maintained for 3 min. Finally, the column was equilibrated with 5% solvent B for 5 min before reuse.

Triple quadrupole mass spectrometry (6490 series, Agilent Technologies) was set up as follows; capillary voltage 2500 V; nozzle voltage 500 V; nebulizer 40 psi; and nitrogen drying gas flow rate 13 L/min. Gas and drying gas temperatures were maintained at 200°C/180°C for neutral and positive lipid analysis and 180°C/180°C for TMSD-reacted lipid analysis. Quantification analysis was performed in MRM mode using computed transitions for each lipid species. Data processing and statistical analysis of individual data obtained by MRM LC/MS data were obtained by Agilent Mass Hunter Workstation Data Acquisition software. The MRM data for target lipids, including m/z of precursor ions, m/z of product ions, and retention time were exported with Qualitative Analysis B.06.00 software (Agilent Technologies). Lipid peaks were assigned by comparing to the retention time of each class internal standard, and Skyline software (MacCoss Lab, University of Washington, Seattle, WA) was used for mining area information of each assigned lipid from replicated raw data. Peak areas were normalized to internal standards and the peak area ratio between PA substrate and the corresponding DG product was calculated for each PA-DG pair. Sums of the PA/DG ratios were compared between the early-onset DGC expressing *TACC2-PPAPDC1A* and tumors without the fusion (n=8) using a one-sample rank sum test.

Supplementary Figures

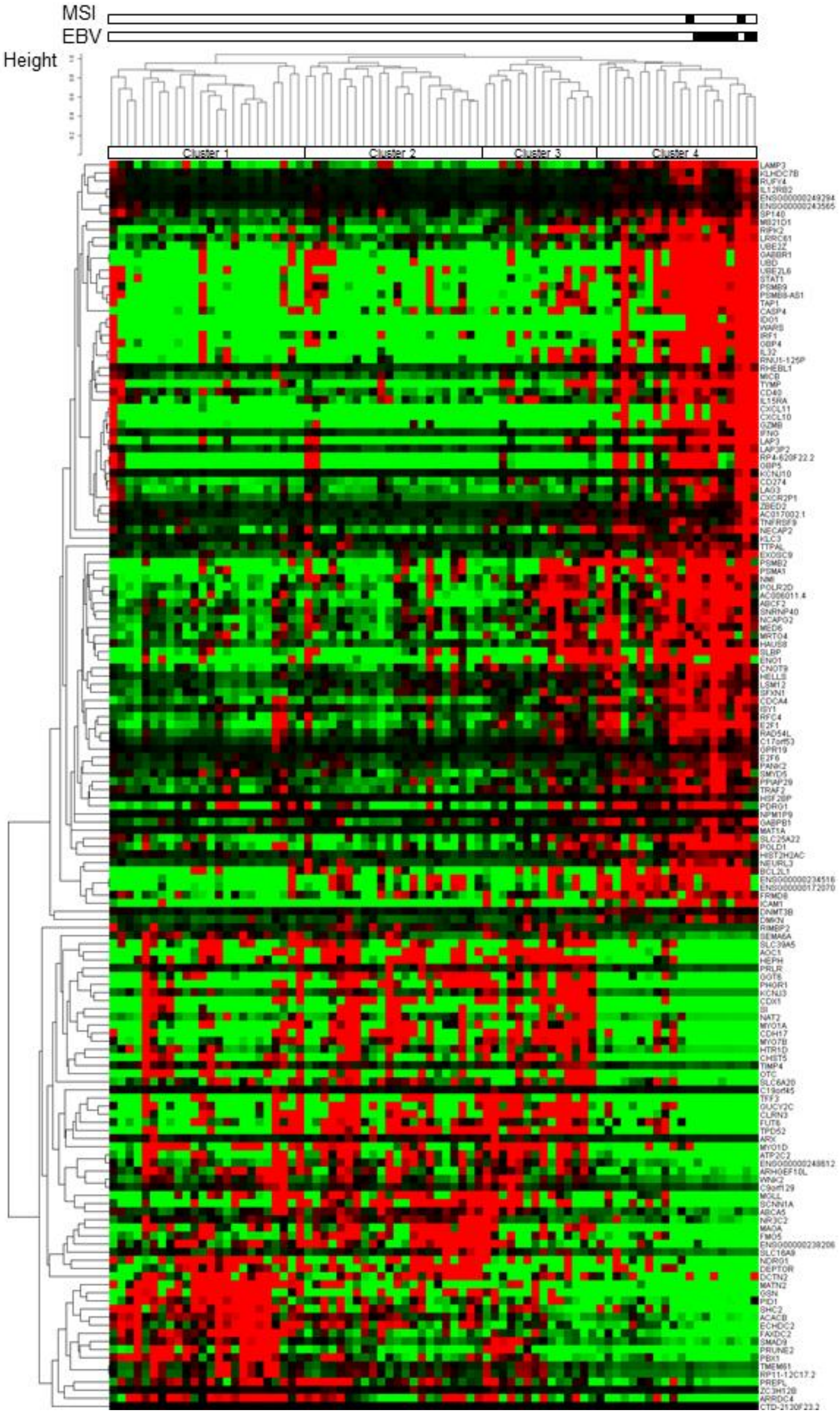

Supplementary Figure 1. Four expression clusters generated by an average linkage hierarchical clustering analysis of 80 DGCs in a discovery dataset using RPKM values for 51,374 genes. Cluster 4 included all microsatellite unstable (MSI; n=2) and Epstein-Barr virus (EBV)-positive tumors (n=7). The heatmap after gene centering is shown. Red, high expression; green, low expression. Immune-related genes (top panel), such as *CD274*, were overexpressed in cluster 4. *H. pylori*-positive tumors were not significantly enriched in Cluster 4 (P=0.44, chi-square)

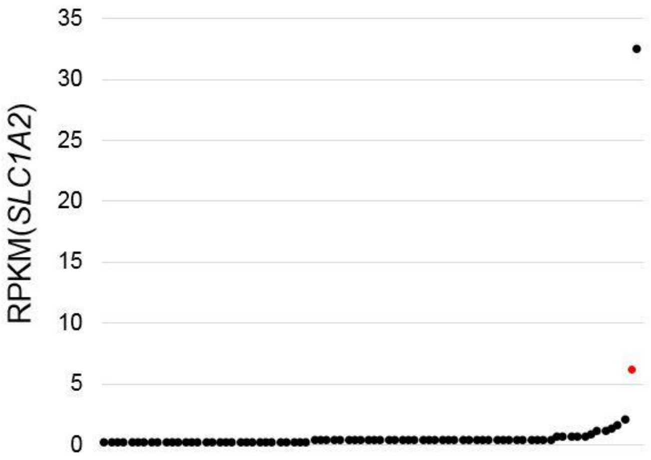

Supplementary Figure 2. Expression level of *SLC1A2* in the tumor containing the fusion (red dot). Sample ordering according to *SLC1A2* expression level.

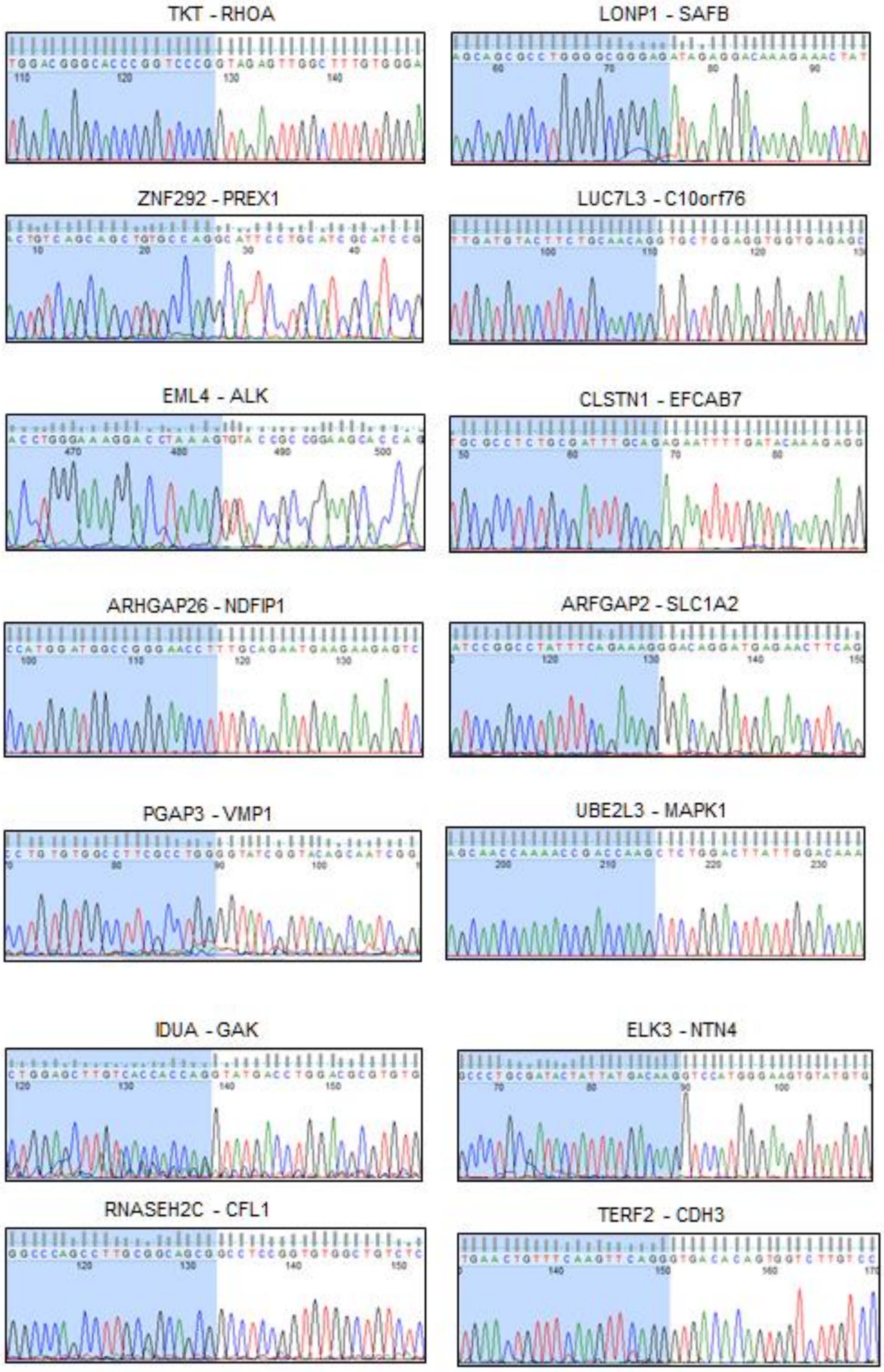
TKT - RHOA
LONP1 - SAFB
ZNF292 - PREX1
LUC7L3 - C10orf76
EML4 - ALK
CLSTN1 - EFCAB7
ARHGAP26 - NDFIP1
ARFGAP2 - SLC1A2
PGAP3 - VMP1
UBE2L3 - MAPK1
IDUA - GAK
ELK3 - NTN4
RNASEH2C - CFL1
TERF2 - CDH3

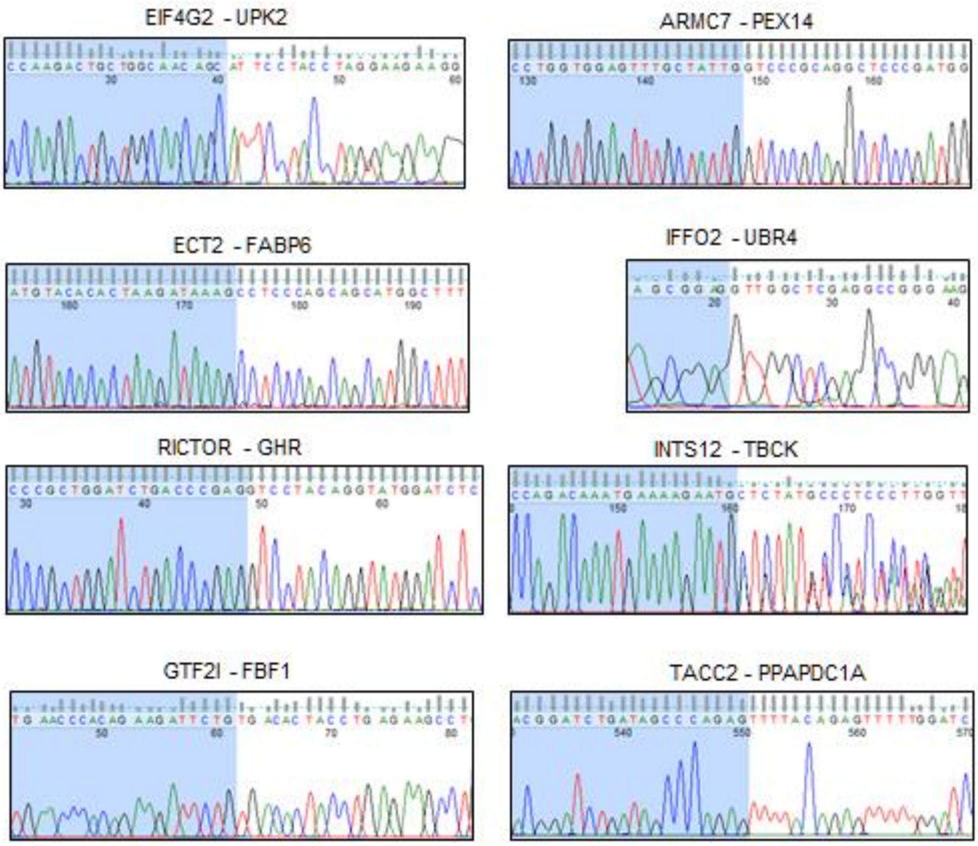

Supplementary Figure 3. RT-PCR capillary sequencing analysis for the validation of recurrent gene fusions in a discovery dataset.

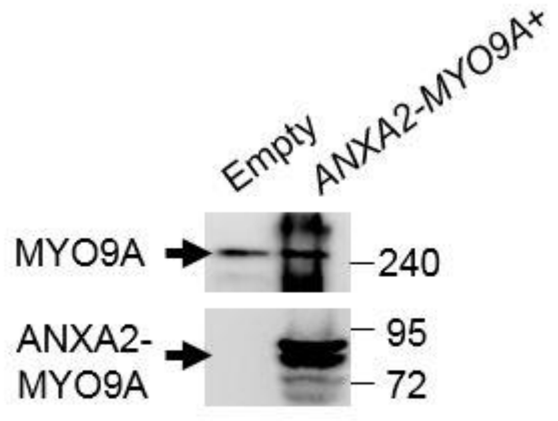

Supplementary Figure 4. Western blot for MYO9A in 293FT cells following the ectopic expression of empty (left) or *ANXA-MYO9A* fusion (right). Primary antibody used was anti-MYO9A antibody that binds to the c-terminal portion of MYO9A. The molecular weight of endogenous MYO9A is about 292kDa. The molecular weight of fusion protein ANXA2-MYO9A is estimated 72kDa.

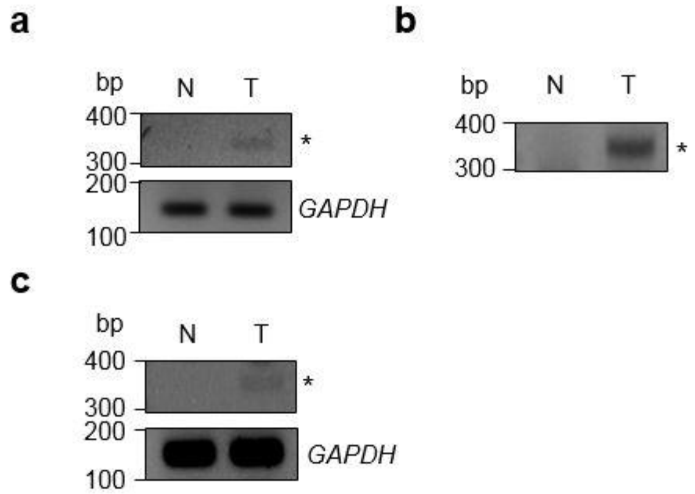

Supplementary Figure 5. RT-PCR and genomic DNA PCR for the *ANXA2-MYO9A* fusion (a) RT-PCR analysis of the early-onset DGC expressing the *ANXA2-MYO9A* fusion (right) and it adjacent normal tissue (left) (b) Genomic DNA PCR analysis of the early-onset DGC expressing the *ANXA2-MYO9A* fusion (right) and it adjacent normal tissue (left) (c) RT-PCR analysis of the late-onset DGC expressing the *ANXA2-MYO9A* fusion (right) and it adjacent normal tissue (left). Asterisk, a band representing the *ANXA2-MYO9A* fusion.

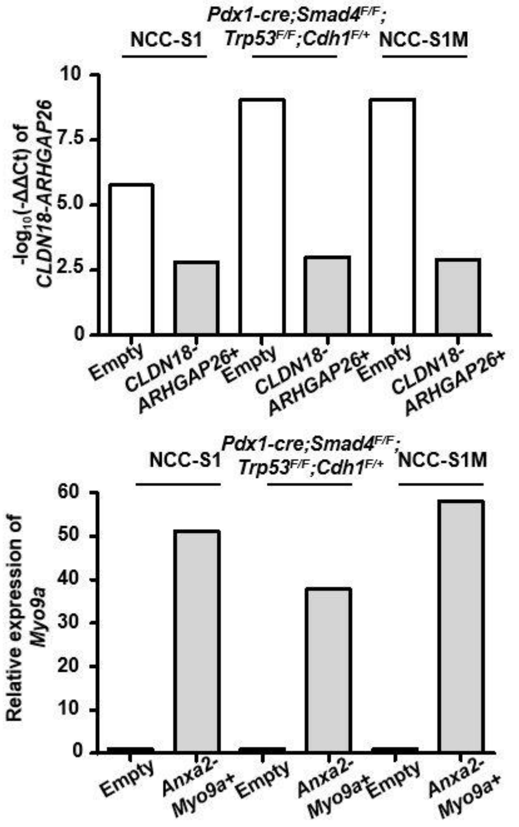

Supplementary Figure 6. Quantitative real-time RT-PCR for *CLDN18-ARHGAP26* and *Myo9a*.

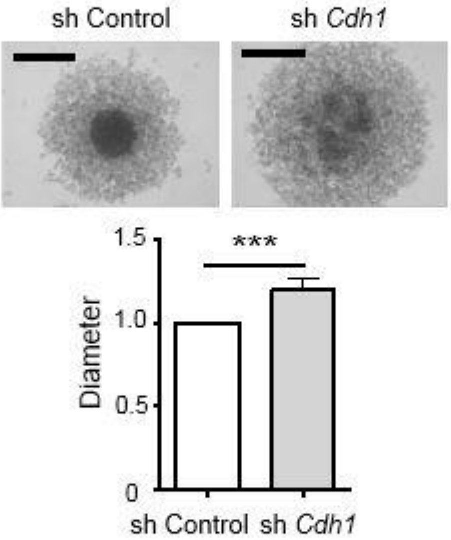

Supplementary Figure 7. Slow aggregation assays of NCC-S1 after gene silencing of *Cdh1*. Top panel, Representative photographs (scale bar=0.5 mm); Bottom panel, relative value of cell aggregate diameter. Average value for three independent experiments. ***P<0.0001, *t*-tests; Error bar, mean ± SEM

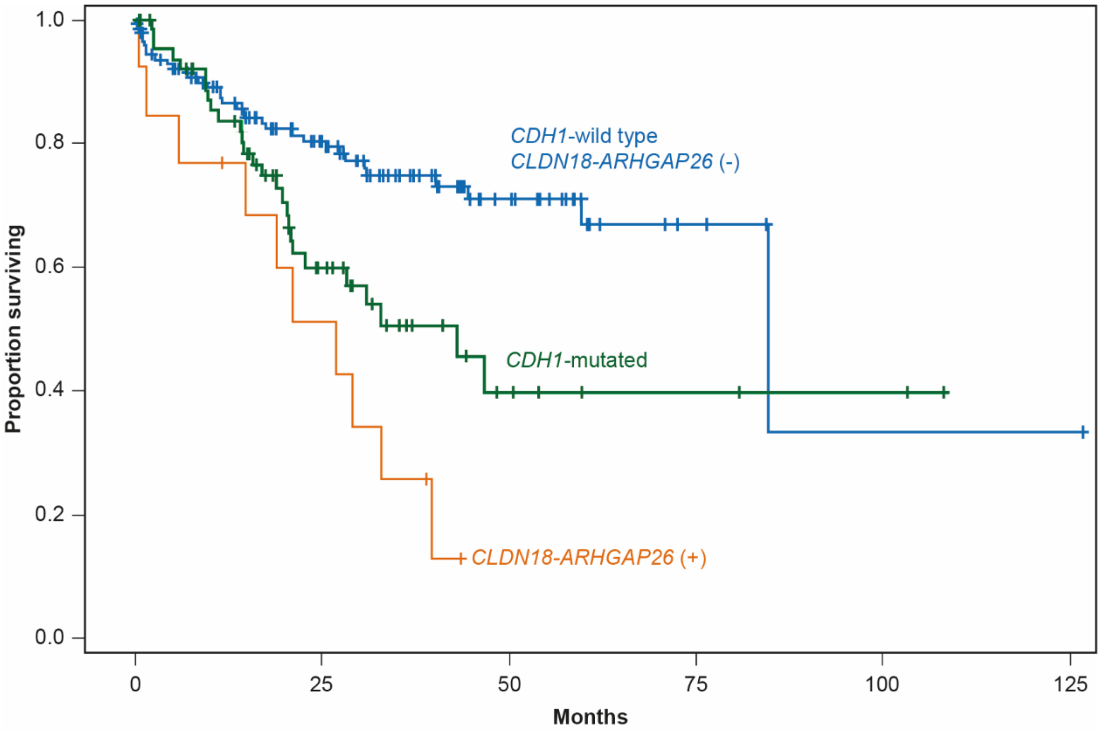

Supplementary Figure 8. Prognostic effects of *CLDN18-ARHGAP26* vs. *CDH1* mutation. Kaplan-Meier curves for overall survival of patients with DGCs expressing the *CLDN18-ARHGAP26* fusion (n=13; shown in red) vs. *CDH1*-mutated DGCs (n=66; shown in green) vs. DGCs that were wild-type for *CDH1* and *CLDN18-ARHGAP26* fusion (n=150; shown in blue). Median survival times were 26.9 months, 43.1 months, and 84.8 months, respectively, for DGCs expressing the *CLDN18-ARHGAP26* fusion, *CDH1*-mutated DGCs, DGCs that were wild-type for *CDH1* and *CLDN18-ARHGAP26* fusion. Tumors that were subject to targeted DNA sequencing analyses (n=229) were included in this survival analysis.

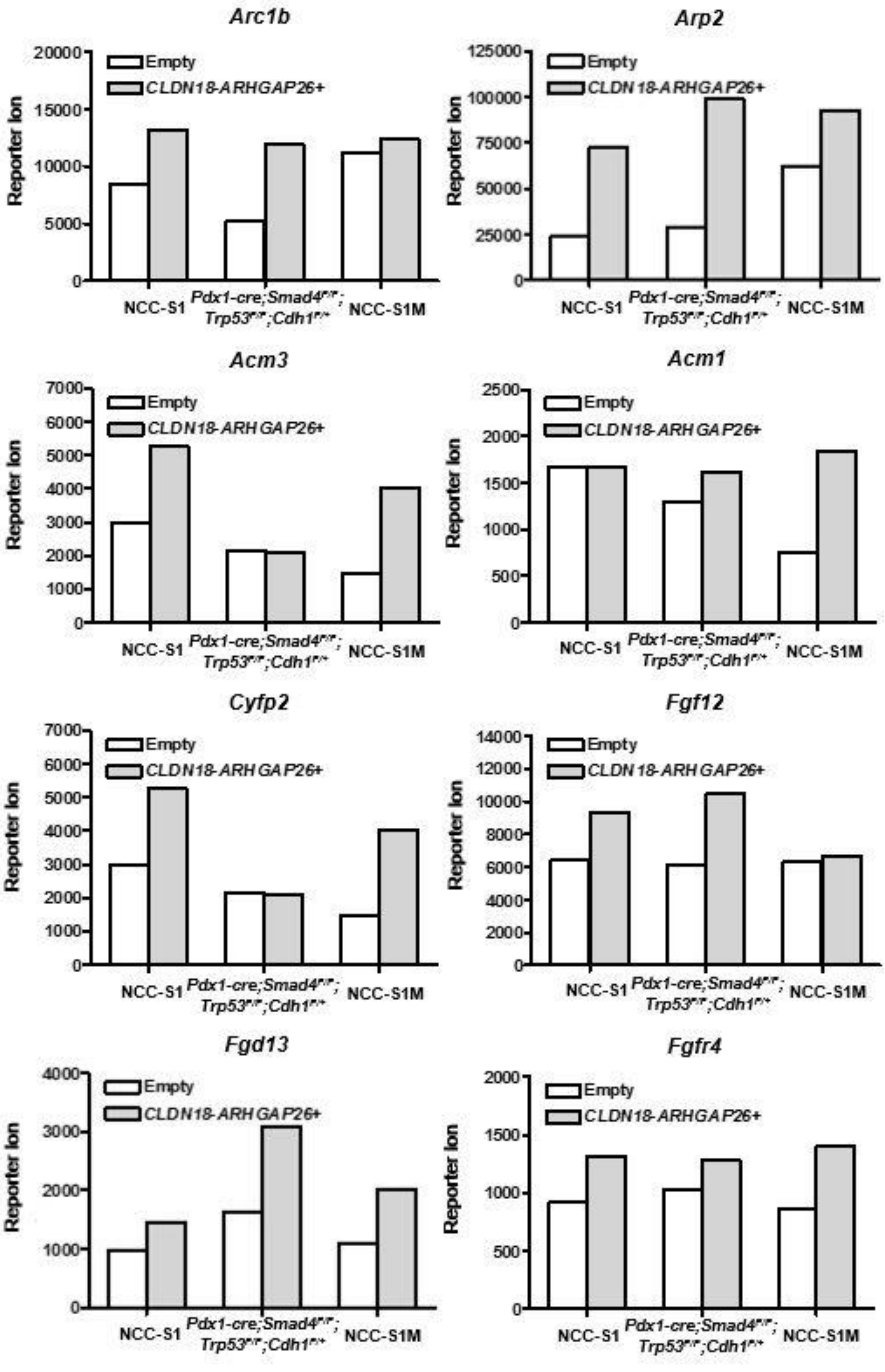
Arc1b
Reporter Ion
20000
15000
10000
5000
0
Empty
CLDN18-ARHGAP26+
NCC-S1
Pdx1-cre;Smad4F/F;
Trp53F/F;Cdh1F/+
NCC-S1M
Arp2
Reporter Ion
125000
100000
75000
50000
25000
0
Empty
CLDN18-ARHGAP26+
NCC-S1
Pdx1-cre;Smad4F/F;
Trp53F/F;Cdh1F/+
NCC-S1M
Acm3
Reporter Ion
7000
6000
5000
4000
3000
2000
1000
0
Empty
CLDN18-ARHGAP26+
NCC-S1
Pdx1-cre;Smad4F/F;
Trp53F/F;Cdh1F/+
NCC-S1M
Acm1
Reporter Ion
2500
2000
1500
1000
500
0
Empty
CLDN18-ARHGAP26+
NCC-S1
Pdx1-cre;Smad4F/F;
Trp53F/F;Cdh1F/+
NCC-S1M
Cyfp2
Reporter Ion
7000
6000
5000
4000
3000
2000
1000
0
Empty
CLDN18-ARHGAP26+
NCC-S1
Pdx1-cre;Smad4F/F;
Trp53F/F;Cdh1F/+
NCC-S1M
Fgf12
Reporter Ion
14000
12000
10000
8000
6000
4000
2000
0
Empty
CLDN18-ARHGAP26+
NCC-S1
Pdx1-cre;Smad4F/F;
Trp53F/F;Cdh1F/+
NCC-S1M
Fgd13
Reporter Ion
4000
3000
2000
1000
0
Empty
CLDN18-ARHGAP26+
NCC-S1
Pdx1-cre;Smad4F/F;
Trp53F/F;Cdh1F/+
NCC-S1M
Fgfr4
Reporter Ion
2000
1500
1000
500
0
Empty
CLDN18-ARHGAP26+
NCC-S1
Pdx1-cre;Smad4F/F;
Trp53F/F;Cdh1F/+
NCC-S1M

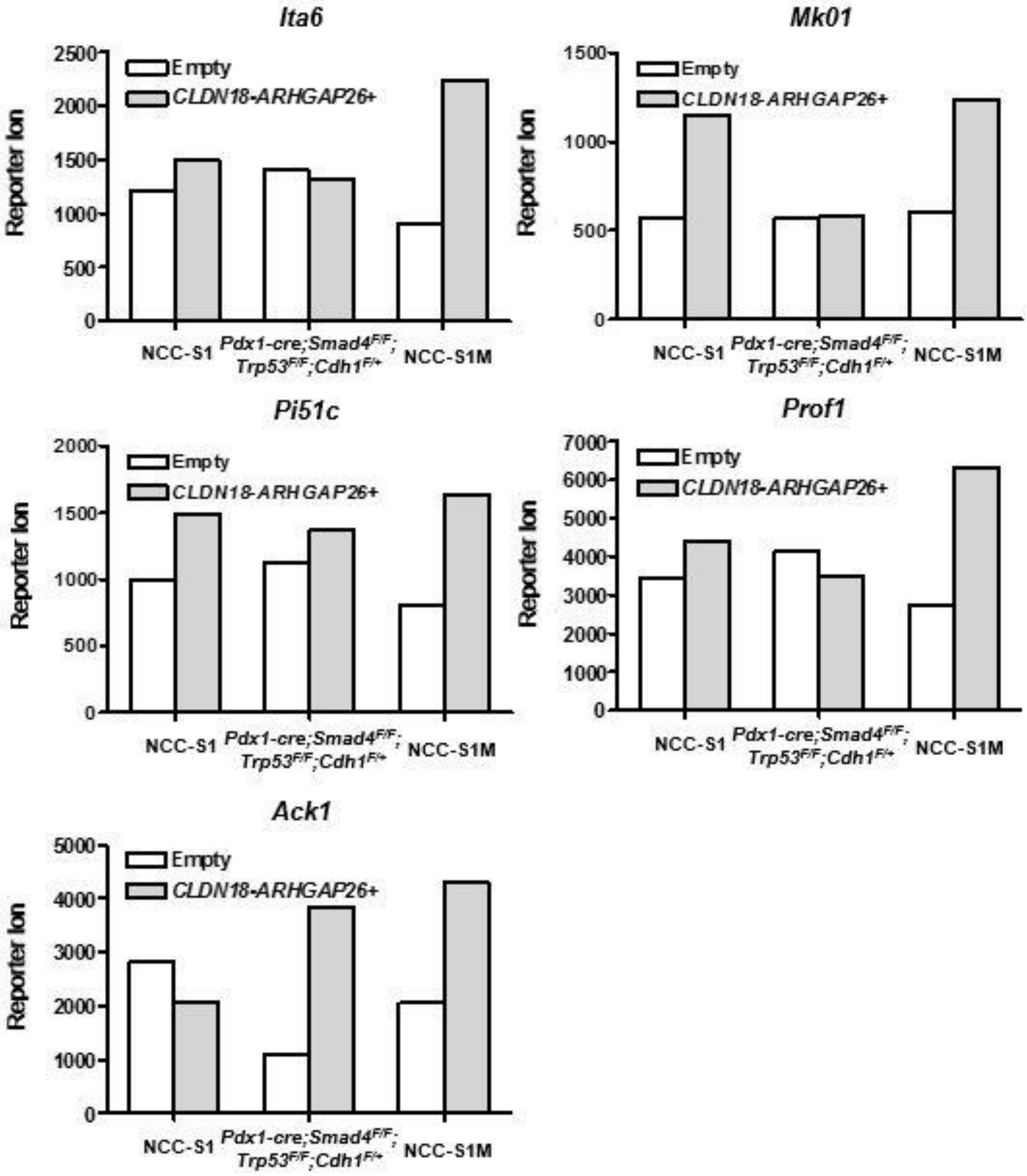

Supplementary Figure 9. Phosphoproteins in *Regulation of actin cytoskeleton* KEGG pathway that were upregulated in mouse gastric cancer cells by the ectopic expression of *CLDN18-ARHGAP26.* Y-axis, reporter ion intensity; white bar, mouse gastric cancer cells expressing an empty vector; gray bar, mouse gastric cancer cells expressing *CLDN18-ARHGAP26.*

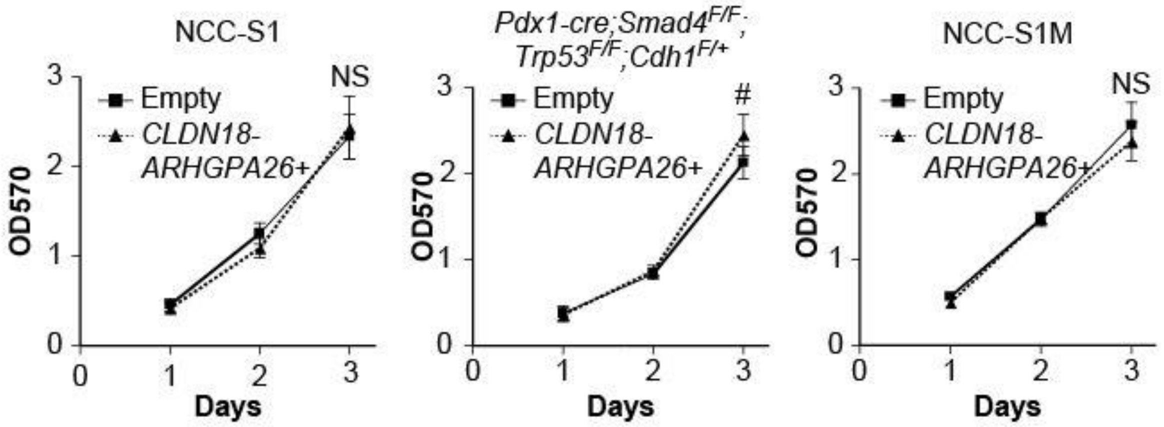

Supplementary Figure 10. MTT assay for the effect of *CLDN18-ARHGAP26* overexpression on *in vitro* proliferation of mouse gastric cancer cells. Mouse gastric cancer cells overexpressing *CLDN18-ARHGAP26* did not differ from those expressing an empty vector in the monolayer growth potential (P=0.40, paired *t*-test). #P<0.05

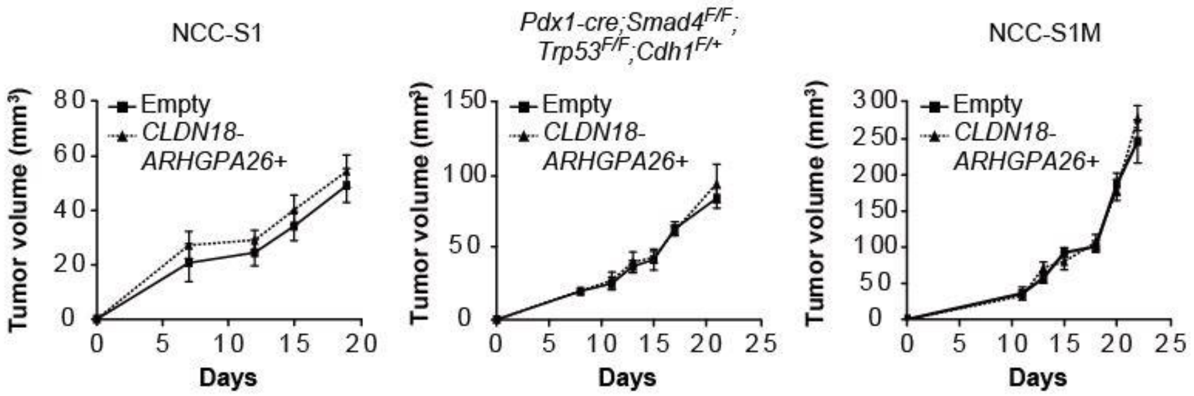

Supplementary Figure 11. *In vivo* growth of mouse gastric cancer cell allografts. Mouse gastric cancer cells overexpressing *CLDN18-ARHGAP26* did not differ from those expressing an empty vector in tumorigenic and growth potentials *in vivo* (P values, 0.07, 0.58, and 0.71, respectively; repeated measure ANOVA). Error bars, mean ± SEM

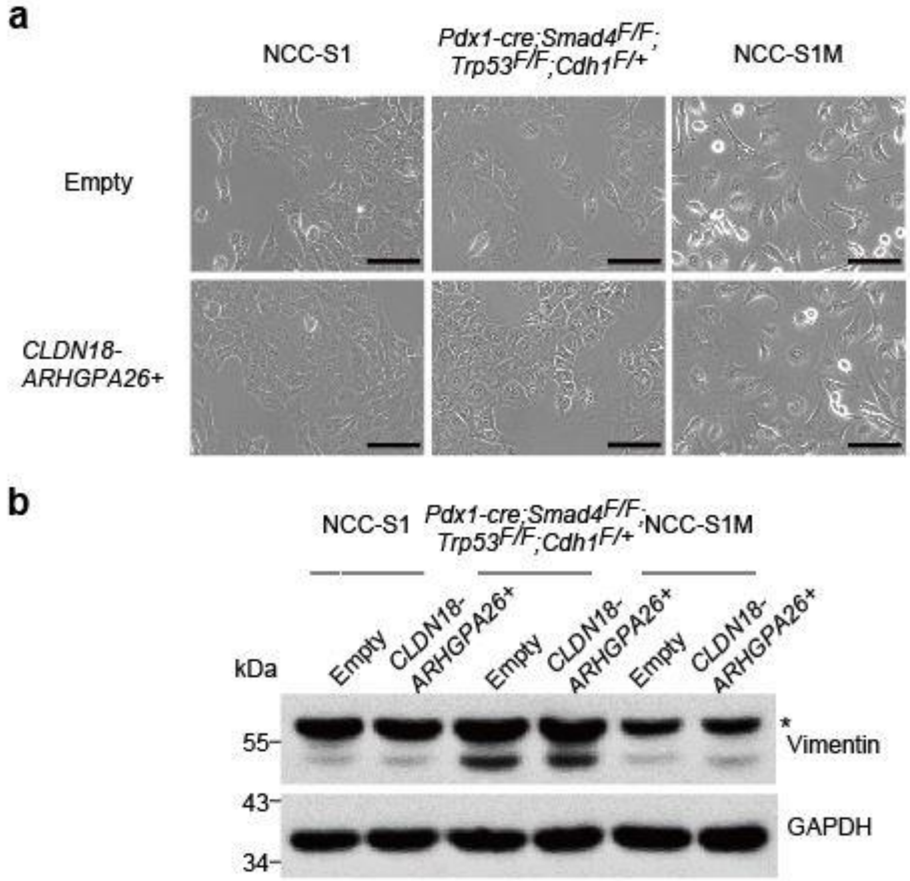

Supplementary Figure 12. Effect of *CLDN18-ARHGAP26* on cell morphology (a) Changes in *in vitro* morphology of mouse gastric cancer cell lines were not prominent following the ectopic expression of *CLDN18-ARHGAP26* (200x). Scale bar=0.1mm (b) Vimentin protein expression did not change after the ectopic expression of *CLDN18-ARHGAP26* in mouse gastric cancer cell lines. Asterisk, a Western blot band representing the Vimentin.

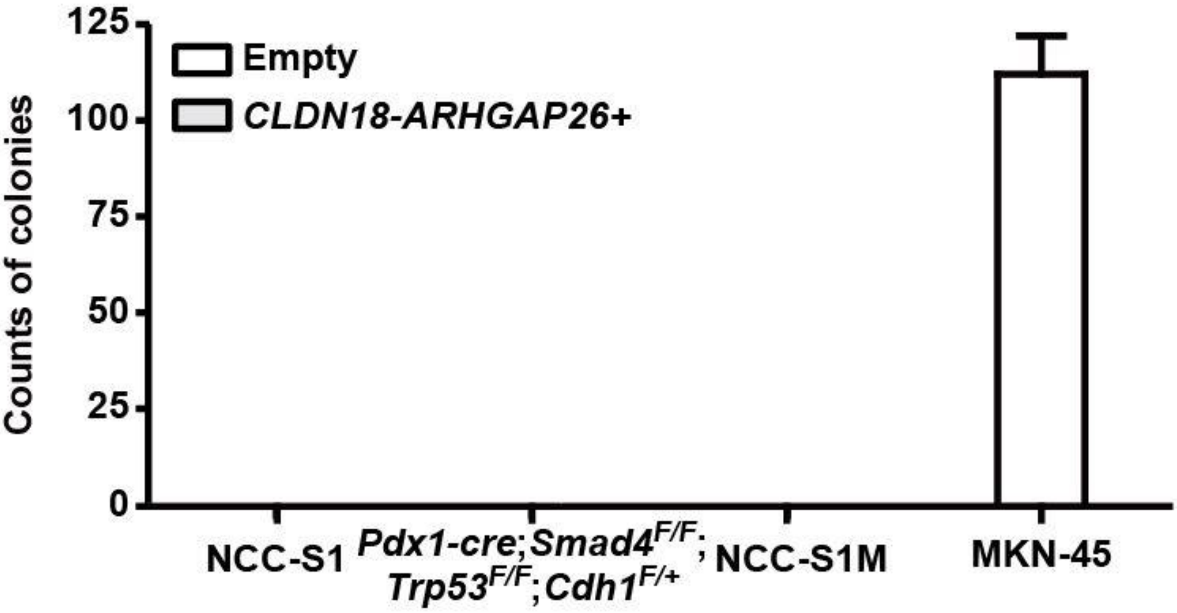

Supplementary Figure 13. Soft agar assay of mouse gastric cancer cell lines after expression of *CLDN18-ARHGAP26* fusion. *CLDN18-ARHGAP26* expression did not promote soft agar colony formation in mouse gastric cancer cells. MKN-45 was used as positive control. Average values for two independent experiments. Error bars, mean ± SEM.

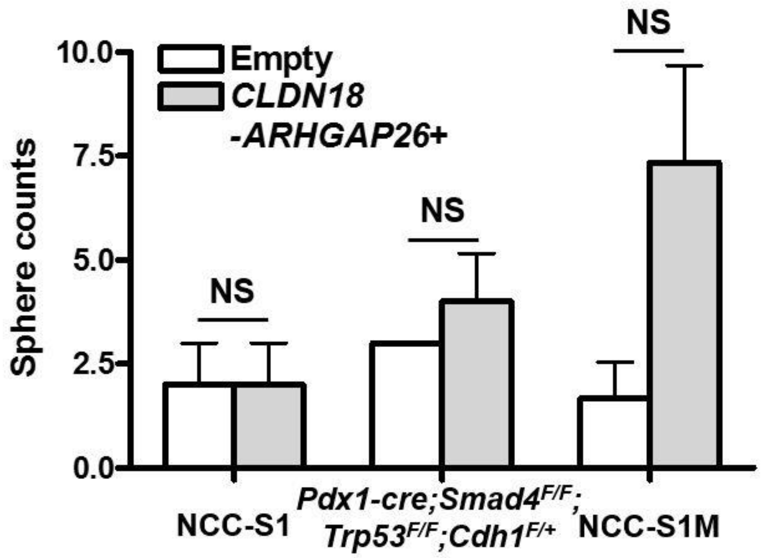

Supplementary Figure 14. Sphere formation assay in mouse gastric cancer cell lines. Ectopic expression of *CLDN18-ARHGAP26* did not significantly promote sphere formation (P=0.094, Wilcoxon signed rank). Average values for at least three independent experiments. Error bars, mean ± SEM.

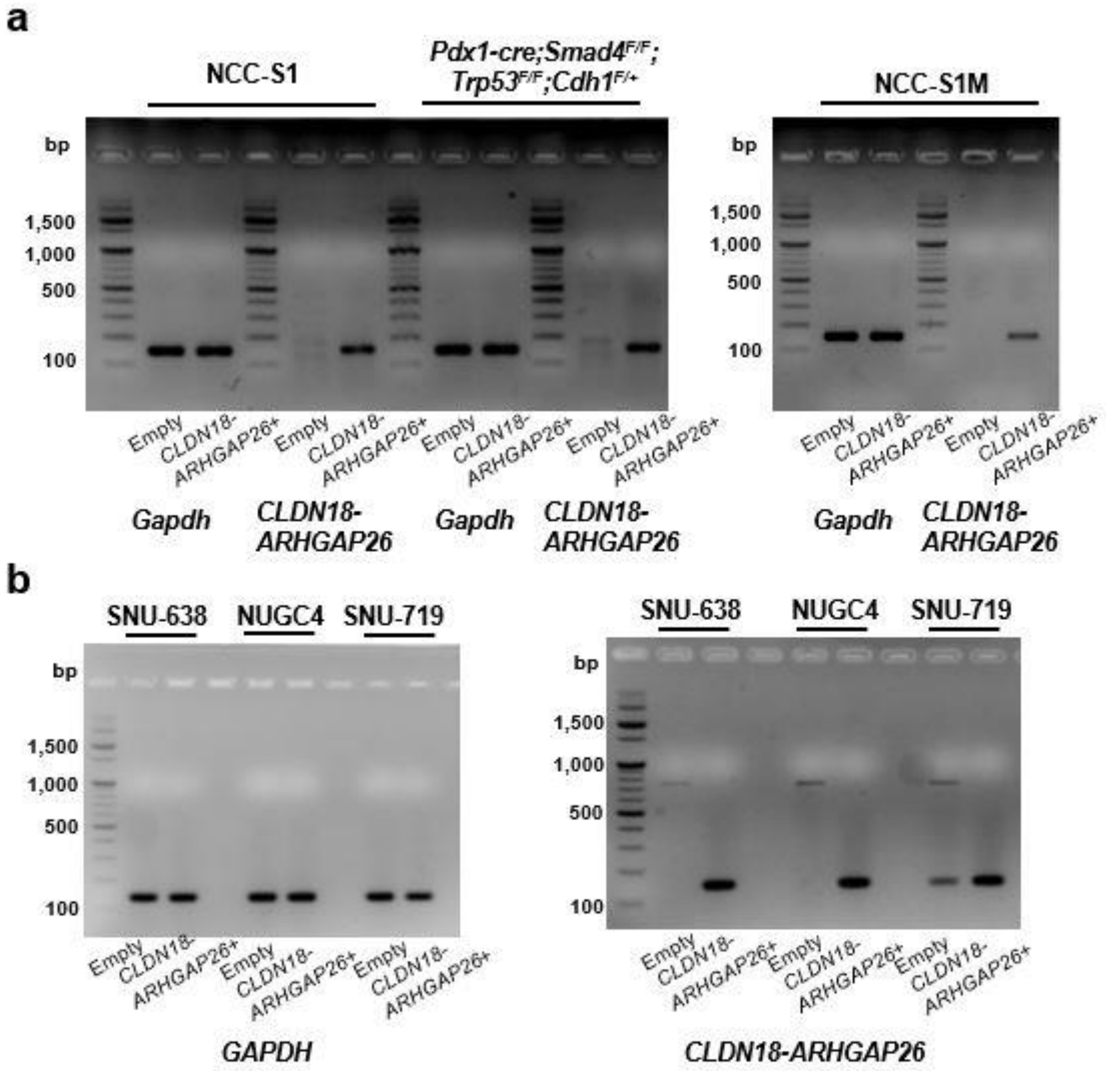

Supplementary Figure 15. Full gel images of *CLDN18-ARHGAP26* RT-PCR (a) Mouse gastric cancer cells (b) Human gastric cancer cells

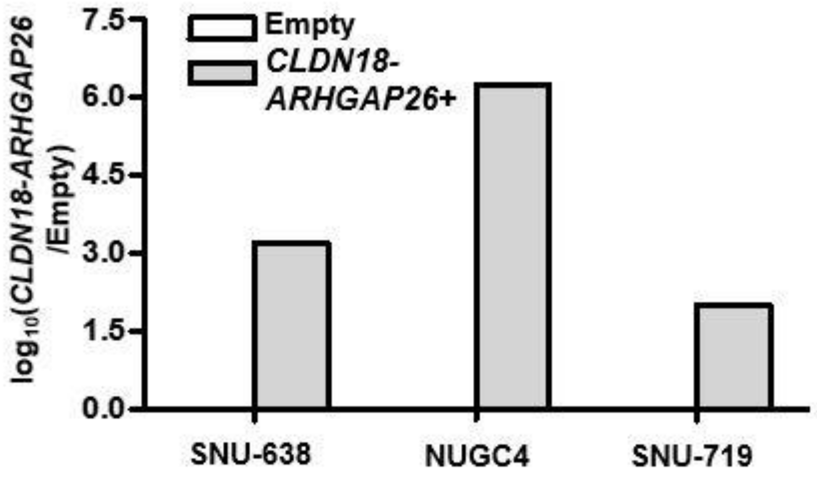

Supplementary Figure 16. *CLDN18-ARHGAP26* quantitative real-time RT-PCR validating overexpression of the *CLDN18-ARHGAP26* fusion in stable transfectants. White bar, cells stably expressing empty vector; gray bar, cells stably expressing *CLDN18-ARHGAP26*.

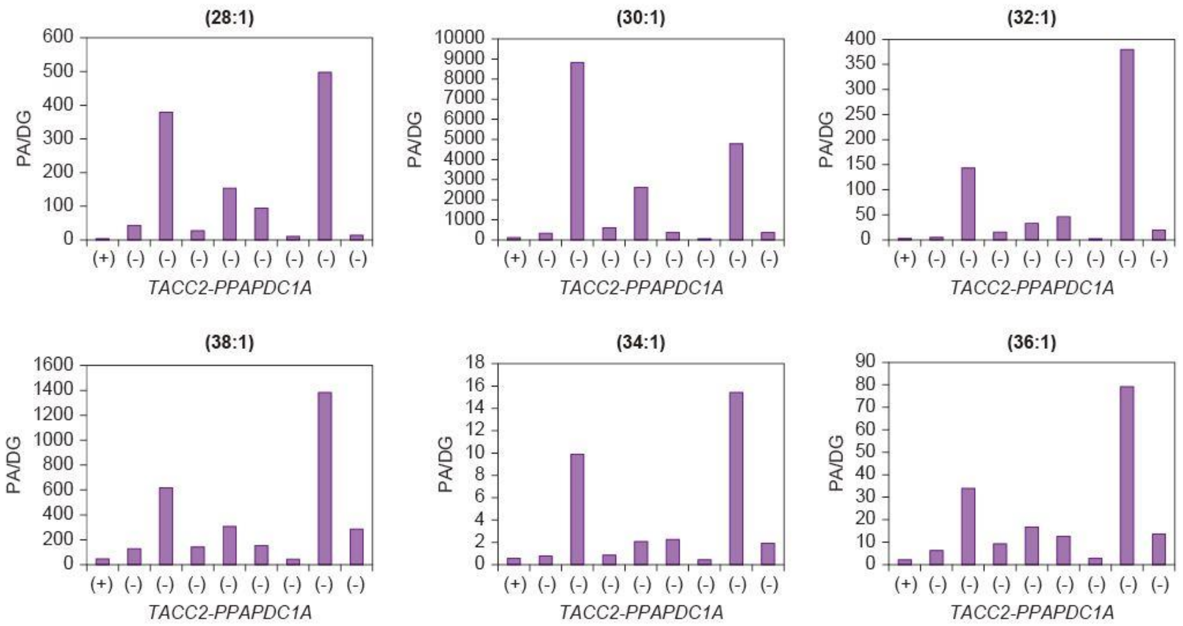

Supplementary Figure 17. The mass spectral peak area ratio between phosphatidic acid (PA) and corresponding diacylglycerol (DG) for each PA-DG pair. Lipidomic profiling was performed on the early-onset DGC expressing *TACC2-PPAPDC1A* (n=1) and eight randomly-selected DGCs without the fusion. Vertical axis denotes the mass spectral peak area ratio between phosphatidic acid (PAs, the substrate of PPAPDC1A) and the corresponding diacylglycerol (DGs, the product of PPAPDC1A) for each PA-DG pair. Horizontal axis denotes tumors. Left, the early-onset DGC with *TACC2-PPAPDC1A* in the discovery set; Right, eight randomly-selected DGCs without *TACC2-PPAPDC1A.* Data for six most abundant lipids are shown.

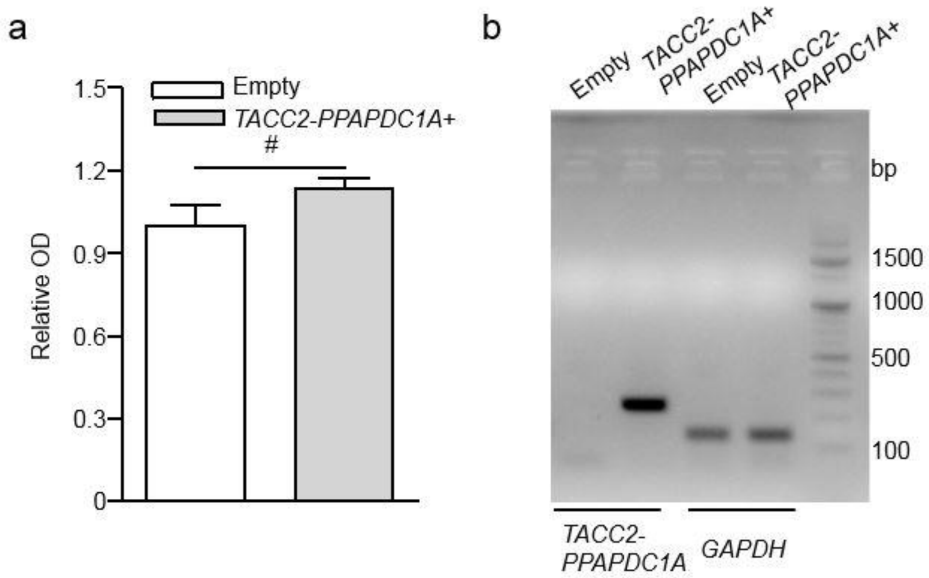

Supplementary Figure 18. Modest effect of transient expression of *TACC2-PPAPDC1A* lentiviral vector on the proliferation of ImSt cells. (a) Relative optical densities (ODs) in MTS assays. Results from two independent experiments. #P=0.043, paired *t*-test; Error bar, mean ± SEM. (b) RT-PCR validation of *TACC2-PPAPDC1A* transgene expression in the ImSt cell line transduced with *TACC2-PPAPDC1A* lentiviral vector (lanes 2 and 4).

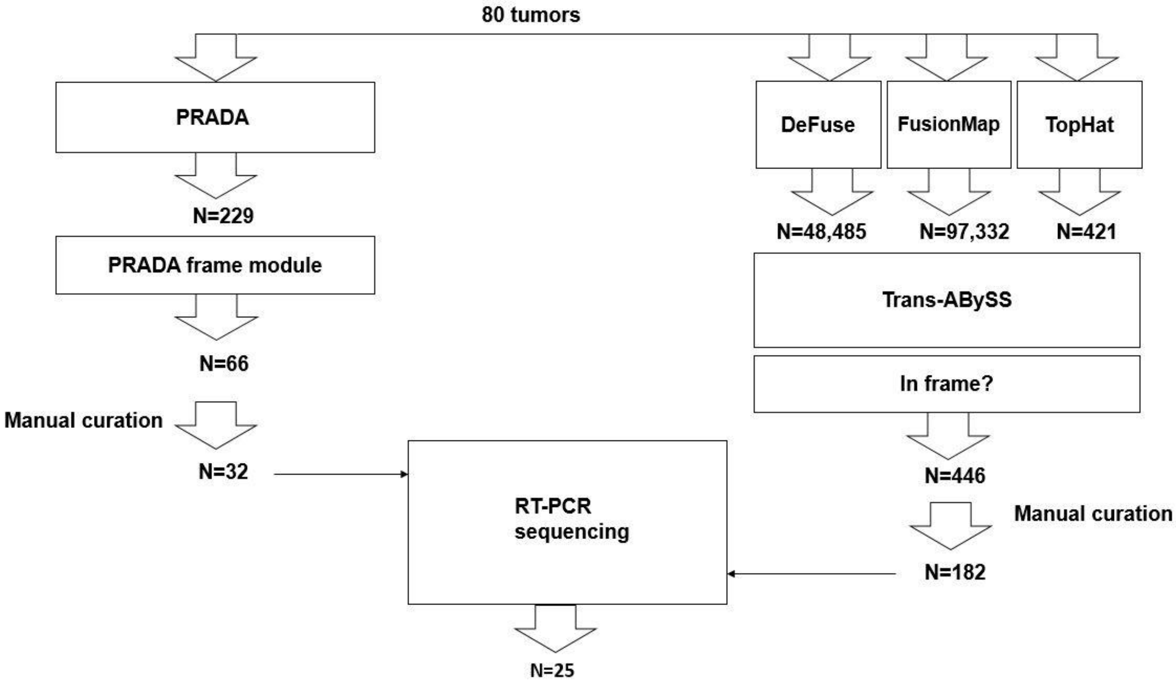

Supplementary Figure 19. Bioinformatics workflow to identify novel gene fusions using RNA sequencing data

Supplementary Tables

Supplementary Table 1. Clinical characteristics of an expanded dataset (n=384)

**Gender**	*n*	%
Male	242	63.0%
Female	142	37.0%

**Age**	*n*	%
Median (range)	53 (20–90)	
Early-onset (≤45)	135	35.2%
Late-onset (≥46)	249	64.8%

**Histology (Lauren)**	*n*	%
Diffuse type	384	100%

**Primary tumor location**	*n*	%
Upper 1/3	71	18.5%
Middle 1/3	126	32.8%
Lower 1/3	164	42.7%
Entire stomach	23	6.0%

**AJCC[1] stage**	*n*	%
IA	14	3.6%
IB	15	3.9%
IIA	32	8.3%
IIB	41	10.7%
IIIA	41	10.7%
IIIB	56	14.6%
IIIC	65	16.9%
IV	120	31.3%

[1]American Joint Committee on Cancer (7th Edition)

Supplementary Table 2. Clinical characteristics of the sequenced dataset (n=305), which was composed of the discovery set (n=80) and DGCs subjected to targeted RNA sequencing (n=225)

**Gender**	*n*	%
Male	185	61.1%
Female	117	38.9%

**Age**	*n*	%
Median (range)	51 (20–90)	
Early-onset (≤45)	115	38.1%
Late-onset (≥46)	187	61.9%

**Histology (Lauren)**	*n*	%
Diffuse type	305	100.00%

**Primary tumor location**	*n*	%
Upper 1/3	61	20.2%
Middle 1/3	106	35.1%
Lower 1/3	124	41.1%
Entire stomach	11	3.6%

**AJCC stage**	*n*	%
IA	13	4.3%
IB	13	4.3%
IIA	26	8.6%
IIB	38	12.6%
IIIA	35	11.6%
IIIB	48	15.9%
IIIC	55	18.2%
IV	74	24.5%

Supplementary Table 3. Top 10 Biocarta pathways enriched in genes that were differentially expressed between Cluster 4, which included all microsatellite unstable (n=2) and EBV-positive tumors (n=7), and the other clusters. These clusters were generated by an average linkage hierarchical clustering analysis of 80 DGCs in a discovery dataset using RPKM values for 51,374 genes

**Biocarta Pathway**	***No* genes**	**P**
B Lymphocyte Cell Surface Molecules	11	0.00001
Caspase Cascade in Apoptosis	22	0.00001
CTL mediated immune response against target cells	13	0.00001
Dendritic cells in regulating TH1 and TH2 Development	17	0.00001
HIV-I Nef: negative effector of Fas and TNF	55	0.00001
IL12 and Stat4 Dependent Signaling Pathway in Th1 Development	19	0.00001
IL-2 Receptor Beta Chain in T cell Activation	37	0.00001
Keratinocyte Differentiation	46	0.00001
CDK Regulation of DNA Replication	18	0.00001
Antigen Processing and Presentation	11	0.00001

P, least-square permutation P value

Supplementary Table 4. Genes that were significantly (P < 10^{6}) overexpressed in Cluster 4, which included all microsatellite unstable and EBV-positive tumors, compared with the other clusters generated by an average linkage hierarchical clustering analysis of 80 DGCs in a discovery dataset using RPKM values for 51,374 genes.

Gene	FDR	RPKM		
		Ratio	Cluster 1_3	Cluster 4
CDCA4	<0.0001	1.8	5.7	10.4
UBE2L6	<0.0001	2	48.3	94.1
RP4-620F22.2	<0.0001	3.8	5.8	22.4
SLC25A22	<0.0001	1.8	6.5	11.8
IDO1	<0.0001	7.1	7.1	50.3
GBP5	<0.0001	4	4.8	19.1
LAMP3	<0.0001	3	3	9.1
PPIAP29	<0.0001	1.9	3.1	5.8
WARS	<0.0001	2.8	56.8	157.5
MB21D1	<0.0001	1.7	3.5	5.9
PSMB2	<0.0001	1.4	31.6	42.8
E2F6	<0.0001	1.4	2.8	4
PSMA1	<0.0001	1.3	55.6	71.9
LRRC61	<0.0001	1.6	3.9	6.4
CNOT9	<0.0001	1.3	10.3	13.6
KLHDC7B	<0.0001	2.5	0.6	1.4
CXCL11	<0.0001	7.1	2.2	16.3
EXOSC9	<0.0001	1.4	5.9	8.1
ISY1	<0.0001	1.2	6	7.4
RHEBL1	<0.0001	2.3	0.8	1.8
NEURL3	<0.0001	4.8	0.3	1.6
ENSG00000234516	<0.0001	1.7	20.3	34.2
GZMB	<0.0001	3.3	4.9	16.7
MED6	<0.0001	1.2	12.3	15.1
POLR2D	0.0001	1.4	9.8	13.7
LSM12	0.0001	1.5	3.2	4.8
UBE2Z	0.0001	1.3	18.9	24.3
MICB	0.0001	2.2	2.3	4.8
TTPAL	0.0001	1.5	3	4.4
MRTO4	0.0001	1.5	12.1	17.7
STAT1	0.0001	2	47.6	97.2
KCNJ10	0.0001	4.3	0.1	0.5
ZBED2	0.0001	3.6	0.3	1
CXCL10	0.0001	5.9	13.9	81.1
HAUS8	0.0001	1.5	4.5	6.7
DNMT3B	0.0001	1.9	0.7	1.4
GABBR1	0.0001	3	13.6	41.1
LAP3	0.0001	1.7	32.6	55
IL12RB2	0.0001	2.8	0.3	0.7
ABCF2	0.0001	1.4	10.9	14.9
SNRNP40	0.0001	1.3	8	10.4
ENSG00000172070	0.0001	1.7	14.7	24.5
IRF1	0.0001	1.9	13.5	25.2
ENSG00000249294	0.0001	2.5	0.2	0.4
LAP3P2	0.0001	1.9	1.7	3.2
RUFY4	0.0001	3.6	0.2	0.7
ENSG00000243565	0.0001	2.3	0.5	1.1
GBP4	0.0001	2.5	11.1	27.7
RFC4	0.0001	1.6	5.7	9.3
IFNG	0.0001	4.3	0.3	1.3
HELLS	0.0001	2	1.5	3
IL32	0.0001	1.9	68.2	132.2
RIPK2	0.0001	1.6	8.7	13.8
AC006011.4	0.0001	1.4	14.4	20.3
HSF2BP	0.0001	2.2	0.2	0.5
SLBP	0.0001	1.4	23	31.4
RNU1-125P	0.0001	2.2	6.7	14.7
UBD	0.0001	4.5	67.4	308
TYMP	0.0001	2	34.7	67.4
PANK2	0.0001	1.3	5.2	6.6
NMI	0.0001	1.4	10.5	15.1
E2F1	0.0001	1.9	4.7	8.9
NPM1P9	0.0001	2.7	0.1	0.3
PSMB9	0.0001	1.9	15.5	29.3
NECAP2	0.0002	1.3	12.4	15.9
CXCR2P1	0.0002	5.9	0.3	1.9
CASP4	0.0002	1.4	26.7	38.6
CD40	0.0002	1.9	3.4	6.4
HIST2H2AC	0.0002	2	0.9	1.8
PDRG1	0.0002	1.4	11.8	16.9
SMYD5	0.0002	1.4	6.3	8.5
DMKN	0.0002	4.3	0.5	1.9
BCL2L1	0.0002	1.5	36.4	55.5
TNFRSF9	0.0002	2.9	0.6	1.8
RAD54L	0.0002	2.1	2	4.2
PSMB8-AS1	0.0002	1.7	36.4	63
FRMD8	0.0002	1.3	12.1	15.9
CD274	0.0002	2.6	2	5
NCAPG2	0.0002	1.9	3.5	6.5
ENO1	0.0002	1.6	149.4	237.4
GPR19	0.0002	2.2	0.3	0.6
TAP1	0.0002	1.9	42.6	81.2
SFXN1	0.0002	1.4	5.1	7.2
AC017002.1	0.0003	2.3	0.6	1.3
GABPB1	0.0003	1.3	6.9	9.1
ICAM1	0.0003	2.3	21.5	49.5
KLC3	0.0003	2.2	0.5	1.1
IL15RA	0.0003	1.5	8.9	13.1
SP140	0.0003	1.8	2.7	4.7
TRAF2	0.0003	1.4	6.4	9.1
C17orf53	0.0003	2	1	1.9
POLD1	0.0003	1.6	7	11
LAG3	0.0003	2.3	2.2	5.2
MAT1A	0.0003	5	0	0.2

Supplementary Table 5. List of 214 fusion candidates subjected for RT-PCR in a discovery set

1. *CLDN18-ARHGAP26*	51. *HSPB8-FOS*	101. *TPP2-PARP4*	151. *DMD-RASGRF2*	201. *SPATA6-NEDD1*
2. *CTNND1-ARHGAP26*	52. *ZNF264-AURKC*	102. *TGA9-NUMA1*	152. *PDCD6-CYSTM1*	202. *VWF-CTNNAL1*
3. *ANXA2-MYO9A*	53. *TMPRSS7-C3orf52*	103. *LAMB3-C7orf73*	153. *PSAP-PGC*	203. *GLIS3-SMARCA2*
4.*TKT-RHOA*	54. *XNDC15-EHMT1*	104. *LTBP4-PPP1R7*	154. *ARID5B-RTKN2*	204. *RPS6-ILK*
5. *ZNF292-PREX1*	55. *MFSD3-LRRC14*	105. *RPL37A-DTX1*	155. *CDK13-FAM189B*	205. *CACNB2-PIP4K2A*
6. *ECT2-FABP6*	56. *SLC35A3-HIAT1*	106. *RPS4X-PAX9*	156. *MYO5C-FAF1*	206. *UNC50-MBD4*
7. *EML4-ALK*	57. *MSMB-NCOA4*	107. *SDC4-SCP2*	157. *PBX3-TBCD*	206. *EPS15-SMARCA1*
8. *PGAP3-VMP1*	58. *CELF2-PAM*	108. *TRIO-TCF7L1*	158. *PGA3-BCAR1*	207. *ZNF264-AURKC*
9. *TACC2-PPAPDC1A*	59. *TKT-RHOA*	109. *BDH1-CEACAM5*	159. *PKHD1-NBR1*	208. *PLEKHH3-SRC*
10. *LONP1-SAFB*	60. *ARHGAP26-CLDN18*	110. *C1R-TNFRSF21*	160. *CHPF2-WWC2*	209. *PRELID2-LRBA*
11. *LUC7L3-C10orf76*	61. *QSOX2-CARM1*	111. *CCDC85B-ABCA2*	161. *FBXO21-BCAS2*	210. *ING3-DAXX*
12. *CLSTN1-EFCAB7*	62. *SKI-ICA1*	112. *CFL1-LONRF3*	162. *KIF1C-GDF15*	211. *ARID1B-TNFRSF11B*
13. *ARFGAP2-SLC1A2*	63. *SMG6-MYO15A*	113. *COL1A1-HSP90AB1*	163. *LGALS3BP-TM9SF3*	212. *PDE8A-ARO*
14. *TERF2-CDH3*	64. *SNRNP40-MAP2K5*	114. *CYFIP1-CALML4*	164. *MRPS25-BRD2*	213. *SULF2-PDCD2*
15. *GTF2I-FBF1*	65. *MTUS1-ASAH1*	115. *ICK-TRIM47*	165. *SAMD5-SASH1*	214. *VSIG2-PGAP3*
16. *ARMC7-PEX14*	66. *PHC3-SMARCE1*	116. *IFRD2-LSR*	166. *VPS41-USP46*	
17. *IFFO2-UBR4*	67. *PHC3-ACACA*	117. *LCN2-LMF2*	167. *KBTBD4-TERF2*	
18. *INTS12-TBCK*	68. *ACACA-PLXDC1*	118. *LGALS4-RPLP2*	168. *PRAP1-HNRNPR*	
19. *UBE2L3-MAPK1*	69. *ACACA-PRKCI*	119. *LGMN-CTSB*	169. *PRDX5-MAPK13*	
20. *ELK3-NTN4*	70. *ACACA-RPL23*	120. *RPL8-MICALL1*	170. *PRSS3-CSF1R*	
21. *EIF4G2-UPK2*	71. *TBL1XR1-HDAC9*	121. *RPS12-LMNA*	171. *DDX5-SFXN4*	
22. *RICTOR-GHR*	72. *CPM-LYZ*	122. *RPS4X-TOMM40L*	172. *LIF-SPG11*	
23. *RNASEH2C-CFL1*	73. *CCT2-NUP107*	123. *S100A14-SEC23A*	173. *NUMB-PDLIM5*	
24. *ARHGAP26-NDFIP1*	74. *ZNHIT2-JMJD1C*	124. *SEPT9-MYSM1*	174. *S100A6-RPS6KB2*	
25. *IDUA-GAK*	75. *YWHAZ-ATAD1*	125. *SLCO2A1-ATP5C1*	175. *ARID1B-PARK2*	
26. *SDC4-SPINT3*	76. *USP8-PSMD13*	126. *SPATA6-NEDD1*	176. *TST-SDF4*	
26. *TSPAN1-POMGNT1*	77. *TTC39B-FYN*	127. *TMEM165-DDX47*	177. *KDM4C-FANCL*	
27. *C4ORF3-FABP2*	78. *TNKS1BP-ARHGAP12*	128. *TMX2-RBM39*	178. *NCK1-RAB5A*	
28. *SIDT2-TAGLN*	79. *TMEM208-LGALS3*	129. *TRPM2-CARKD*	179. *IL32-FOXQ1*	
29. *RABGGTA-DHRS1*	80. *SPTBN1-CKS2*	130. *C1S-SH3BGRL*	180. *CAPN5-CDC123*	
30. *LDLRAD2-HSPG2*	81. *SPINT1-PTBP1*	131. *DCTN2-PRSS22*	181. *HNRNPA3-FLNA*	
31. *TMEM165- CLOCK*	82. *ARID1A-C1QB*	132. *DDX5-SLC35A2*	182. *DROSHA-EEF2*	
32. *ADD1-TC2N*	83. *JUND-CTSZ*	133. *FBLN2-RPL13*	183. *RP2-HNRNPA1*	
33. *IGK-CHD3*	84. *CD2AP-TNFRSF21*	134. *GLI4-HLA*	184. *EZR-CD74*	
34. *TMED7-HSP90AA1*	85. *ACTN1-PDLIM2*	135. *RCBTB1-TBEM66*	185. *NOC4L-GNAS*	
35. *PRKG1-RACGAP1*	86. *ASCC3-PPM1A*	136. *PRR12-RCN3*	186. *SLC1A2-YY1*	
36. *ZNF833P-HNRNPA1*	87. *SMDT1-SKI*	137. *AGBL2-FNBP4*	187. *MGST2-SETD7*	
37. *CARD10-RREB1*	88. *CDC14B-KLHL2*	138. *FLNA-MMP2*	188. *DYRK1B-SQSTM1*	
38. *SCRN1-RABGAP1*	89. *CHST11-CMTM7*	139. *PKD1-NUP85*	189. *ZC3H7B-TRIRAP1*	
39. *NPIPL1-SULT1A3*	90. *CSK-FAM129B*	140. *SLC45A4-XRCC5*	190. *TAF2-SMG1*	
40. *GGTLC2-GGT1*	91. *EEF2-MUC13*	141. *WASL-SND1*	191. *GAS5-ARF6*	
41. *RPL13-COL18A1*	92. *FARSB-TXN*	142. *HTRA1-DOCK1*	192. *CABYR-BACL2A10*	
42. *CTSB-IFT74*	93. *CDC37-S100P*	143. *HIST1H2BG-AGR2*	193. *SEPN1-IDH2*	
43. *FAM192A-SKIDA1*	94. *CHD4-GNL3*	144. *APBB1IP-MAP2K1*	194. *CTSB-VMP1*	
44. *SMARCE1-PHC3*	95. *DMBT1-LCN2*	145. *UCHL5-ETFA*	195. *HPGD-JUNB*	
45. *TAP2-TAP1*	96. *FN1-ZFYVE21*	146. *CDK8-GLUL*	196. *GREM1-VAMP1*	
46. *SAV1-GYPE*	97. *MUC13-TNFRSF25*	147. *PIM1-C7*	197. *DKFZp686O16217-C14orf166*	
47. *ATP2A3-SLC20A2*	98. *MVP-STARD8*	148. *SLC22A3-MUC13*	198. *SCAPER-MECOM*	
48. *RAB5C-DSC2*	99. *PDS5A-FOSL1*	149. *TMEM164-PFN1*	199. *NAP1L-CPSF6*	
50. *LYZ -CCT2*	100. *PHC3-ACACA*	150. *KAZN-MXI1*	200. *ADRBK2-GNAS*	

Supplementary Table 6. DAVID gene ontology analysis (GOTerm_BP_Direct) for 3' partner genes of 25 in-frame fusions identified in a discovery dataset

GOTerm_BP_Direct	Gene	P
apical junction assembly	*FBF1,RHOA*	0.009
actin cytoskeleton organization	*ARHGAP26,CFL1,RHOA*	0.0093
regulation of small GTPase mediated signal transduction	*ARHGAP26,MYO9A,RHOA*	0.01
endoplasmic reticulum organization	*GAK,BMP1*	0.03
signal transduction	*NDFIP1,ARHGAP26,ALK,MAPK1,MYO9A*	0.038
viral process	*MAPK1,RHOA,UBR4*	0.044

Supplementary Table 7. Prevalence of recurrent RhoGAP domain-containing gene fusions according to the *H. pylori* status

CLDN18-ARHGAP26

	H. pylori (+)	*H. pylori* (-)	P*
CLDN18-ARHGAP26 (+)	7(5.3%)	2(1%)	
CLDN18-ARHGAP26 (-)	125(94.7%)	192(99%)	0.034

CTNND1-ARHGAP26

	H. pylori (+)	*H. pylori* (-)	P*
CTNND1-ARHGAP26 (+)	2(1.5%)	0(0%)	
CTNND1-ARHGAP26 (-)	130(98.5%)	194(100%)	0.16

ANXA2-MYO9A

	H. pylori (+)	*H. pylori* (-)	P*
ANXA2-MYO9A (+)	0(0%)	2(1%)	
ANXA2-MYO9A (-)	132(100%)	192(99%)	0.52

*P for Fisher's exact test

Supplementary Table 8. *In vivo* tumorigenic potentials in flank subcutaneous tissue 5 days after injection of *Pdx1-cre; Smad4F$^{/F}$; Trp53F$^{/F}$; Cdh1F$^{/+}$* that stably express either empty or fusion constructs

Pdx1-cre;Smad4$^{F/F}$;Trp53$^{F/F}$;Cdh1$^{F/+}$

Group	Tumor incidence	P*
Empty vector	80% (4 of 5)	
CLDN18-ARHGAP26 +	80% (4 of 5)	NS
Anxa2-Myo9a +	80% (4 of 5)	NS

*P for chi-square (compared with empty vector)

Supplementary Table 9. Chromosomal breakpoints of in-frame gene fusions identified in a discovery dataset

5' gene	3' gene	Chromosomal breakpoints
CTNND1	*ARHGAP26*	g.chr11:57,578,103 – g.chr5:142,358,707
ANXA2	*MYO9A*	g.chr15:60,656,550 – g.chr15:72,157,966
GTF2I	*FBF1*	g.chr7:74,122,062 – g.chr17:73,930,977
ECT2	*FABP6*	g.chr3:172,494,736 – g.chr5:159,641,965
INTS12	*TBCK*	g.chr4:106,609,441 – g.chr4:107,128,316
IFFO2	*UBR4*	g.chr1:19,252,458 – g.chr1:19,413,172

Supplementary Table 10. MYO9A immunostaining in DGCs expressing *ANXA2-MYO9A* fusion and randomly-selected, *ANXA2-MYO9A*-negative tumors

	**Grade 0 (-)**	**Grade 1 (+)**	**Grade 2 (++)**	***P**
ANXA2-MYO9A-positive (n=2)	0	1 (50%)	1 (50%)	
ANXA2-MYO9A-negative (n=8)	7 (87.5%)	1 (12.5%)	0	0.013

*P for Cochran-Mantel-Haenszel test

Supplementary Table 11. Cox proportional hazards regression analysis of *CLDN18-ARHGAP26* for overall survival of DGC patients with targeted DNA sequencing data (n=229)

**Variable**	**P**	**HR[1] (95% CI)**
CLDN18-ARHGAP26	0.0001	4.0 (2.0-8.2)
CDH1 mutation	0.0071	2.0 (1.2-3.4)

[1]HR, hazard ratio

Supplementary Table 12. Cox proportional hazards regression analysis of all RhoGAP domain-containing fusions for overall survival of DGC patients with targeted DNA sequencing data (n=229)

**Variable**	**P**	**HR[1] (95% CI)**
RhoGAP domain fusion	0.001	3.1 (1.5-6.1)
CDH1 mutation	0.008	2.0 (1.2-3.3)

[1]HR, hazard ratio

Supplementary Table 13. DAVID KEGG pathways significantly enriched in top 500 phosphoproteins that were upregulated by *CLDN18-ARHGAP26*. The average reporter ion intensity ratio (between cells overexpressing *CLDN18-ARHGAP26* and those expressing an empty vector) was determined for each phosphoprotein among NCC-S1 cells, NCC-S1M cells, and *Pdx1-cre;Smad4*$^{F/F}$*;Trp53*$^{F/F}$*;Cdh1*$^{F/+}$ cells. Phosphoproteins were then ranked by the average reporter ion intensity ratio, and top 500 phosphoproteins (of 7,944 non-redundant phosphoproteins identified) were then analyzed for DAVID pathway analysis. *Regulation of actin cytoskeleton* was one of significantly enriched pathways.

**KEGG pathway**	**P**
Metabolic pathways	0.03
Inositol phosphate metabolism	0.011
Phosphatidylinositol signaling system	0.011
Regulation of actin cytoskeleton	0.011
Cell adhesion molecules	0.013
Carbon metabolism	0.018
Glycolysis / Gluconeogenesis	0.018
Calcium signaling pathway	0.024
Biosynthesis of antibiotics	0.027
Glycerolipid metabolism	0.045
Toxoplasmosis	0.047

Supplementary Table 14. Cox regression survival analysis of *TACC2-PPAPDC1A* in 172 DGCs with available SNP6.0 array and DNA sequencing data.

Variable	P	HR[1] (95% CI)
TACC2-PPAPDC1A	0.001	7.1 (2.1-23.6)
10a26.1 amplification	0.01	1.6 (1.1-2.2)
CDH1 mutations	0.26	1.4 (0.8-2.4)

[1]HR, hazard ratio

Supplementary Table 15. RT-PCR positivity of RhoGAP domain-containing fusions according to the histology. Intestinal type gastric cancer tissue samples were collected from 150 Korean patients with gastric cancer (72.7% male; a median age of 66 years (range, 43–86); 33.3% stage IV).

	**Diffuse type (expanded dataset; n=384)**	**Intestinal type (n=150)**	***P**
CLDN18-ARHGAP26 (+)	13 (3.4%)	1 (0.7%)	
CTNND1-ARHGAP26 (+)	2 (0.5%)	0	
ANXA2-MYO9A (+)	2 (0.5%)	0	
**Overall fusion positivity**	**17 (4.4%)**	**1 (0.7%)**	**0.03**

*P for chi-square

Supplementary References

1. Schoenfeld, D.A. Sample-size formula for the proportional-hazards regression model. *Biometrics* **39**, 499-503 (1983).
2. Yoon, H.M. *et al.* Is the new seventh AJCC/UICC staging system appropriate for patients with gastric cancer? *J Am Coll Surg* **214**, 88-96 (2012).
3. Li, H. & Durbin, R. Fast and accurate long-read alignment with Burrows-Wheeler transform. *Bioinformatics* **26**, 589-95 (2010).
4. Morin, R. *et al.* Profiling the HeLa S3 transcriptome using randomly primed cDNA and massively parallel short-read sequencing. *Biotechniques* **45**, 81-94 (2008).
5. Torres-Garcia, W. *et al.* PRADA: pipeline for RNA sequencing data analysis. *Bioinformatics* **30**, 2224-6 (2014).
6. Cancer Genome Atlas Research, N. Comprehensive molecular characterization of clear cell renal cell carcinoma. *Nature* **499**, 43-9 (2013).
7. McPherson, A. *et al.* deFuse: an algorithm for gene fusion discovery in tumor RNA-Seq data. *PLoS Comput Biol* **7**, e1001138 (2011).
8. Ge, H. *et al.* FusionMap: detecting fusion genes from next-generation sequencing data at base-pair resolution. *Bioinformatics* **27**, 1922-8 (2011).
9. Kim, D. & Salzberg, S.L. TopHat-Fusion: an algorithm for discovery of novel fusion transcripts. *Genome Biol* **12**, R72 (2011).
10. Robertson, G. *et al.* De novo assembly and analysis of RNA-seq data. *Nat Methods* **7**, 909-12 (2010).
11. Simpson, J.T. *et al.* ABySS: a parallel assembler for short read sequence data. *Genome Res* **19**, 1117-23 (2009).
12. Li, B. & Dewey, C.N. RSEM: accurate transcript quantification from RNA-Seq data with or without a reference genome. *BMC Bioinformatics* **12**, 323 (2011).
13. Yan, F. *et al.* Epidermal growth factor receptor activation protects gastric epithelial cells from Helicobacter pylori-induced apoptosis. *Gastroenterology* **136**, 1297–1307 (2009)
14. Cho, S.Y. *et al.* Sporadic Early-Onset Diffuse Gastric Cancers Have High Frequency of Somatic CDH1 Alterations, but Low Frequency of Somatic RHOA Mutations Compared With Late-Onset Cancers. *Gastroenterology* **153**, 536-549 e26 (2017).
15. Mok, H. J. et al. Age-associated lipidome changes in metaphase II mouse oocytes. *PLOS One* **11**, E0148577 (2016).
